# Qß Virus-like particle-based vaccine induces robust immunity and protects against tauopathy

**DOI:** 10.1038/s41541-019-0118-4

**Published:** 2019-06-03

**Authors:** Nicole M. Maphis, Julianne Peabody, Erin Crossey, Shanya Jiang, Fadi A. Jamaleddin Ahmad, Maria Alvarez, Soiba Khalid Mansoor, Amanda Yaney, Yirong Yang, Laurel O. Sillerud, Colin M. Wilson, Reed Selwyn, Jonathan L. Brigman, Judy L. Cannon, David S. Peabody, Bryce Chackerian, Kiran Bhaskar

**Affiliations:** 1 Department of Molecular Genetics and Microbiology, University of New Mexico, Albuquerque, NM 87131 USA; 2 School of Medicine, University of New Mexico, Albuquerque, NM 87131 USA; 3 College of Pharmaceutical Sciences, University of New Mexico, Albuquerque, NM 87131 USA; 4 Department of Neurology, University of New Mexico, Albuquerque, NM 87131 USA; 5 Department of Radiology, University of New Mexico, Albuquerque, NM 87131 USA; 6 Department of Neurosciences, University of New Mexico, Albuquerque, NM 87131 USA; 7 Present Address: Internal Medicine Residency Program, Department of Medicine, John A Burns School of Medicine, University of Hawaii, Honolulu, HI 96813 USA

**Keywords:** Alzheimer's disease, Peptide vaccines

## Abstract

Tauopathies, including frontotemporal dementia (FTD) and Alzheimer’s disease (AD) are progressive neurodegenerative diseases clinically characterized by cognitive decline and could be caused by the aggregation of hyperphosphorylated pathological tau (pTau) as neurofibrillary tangles (NFTs) inside neurons. There is currently no FDA-approved treatment that cures, slows or prevents tauopathies. Current immunotherapy strategies targeting pTau have generated encouraging data but may pose concerns about scalability, affordability, and efficacy. Here, we engineered a virus-like particle (VLP)-based vaccine in which tau peptide, phosphorylated at threonine 181, was linked at high valency to Qß bacteriophage VLPs (pT181-Qß). We demonstrate that vaccination with pT181-Qß is sufficient to induce a robust and long-lived anti-pT181 antibody response in the sera and the brains of both Non-Tg and rTg4510 mice. Only sera from pT181-Qß vaccinated mice are reactive to classical somatodendritic pTau in human FTD and AD post-mortem brain sections. Finally, we demonstrate that pT181-Qß vaccination reduces both soluble and insoluble species of hyperphosphorylated pTau in the hippocampus and cortex, avoids a Th1-mediated pro-inflammatory cell response, prevents hippocampal and corpus callosum atrophy and rescues cognitive dysfunction in a 4-month-old rTg4510 mouse model of FTD. These studies provide a valid scientific premise for the development of VLP-based immunotherapy to target pTau and potentially prevent Alzheimer’s diseases and related tauopathies.

## Tauopathies: Active vaccination of mouse tauopathy

Tauopathies such as fronto-temporal dementia or Alzheimer’s disease are characterized by the accumulation of phosphorylated Tau (pTau) protein into pathogenic neurofibrillary tangles (NFT). Kiran Bhaskar and colleagues at the University of New Mexico investigate the efficacy of an active vaccine approach in the treatment of rTg4510 mice—an aggressive model of tauopathy. Mice receive 3 intramuscular doses of a disease-relevant pTau peptide (pT181) multivalently conjugated to an immunostimulatory bacteriophage virus-like particle (pT181-Qß). Vaccination induces high titers of anti-pTau—stable to at least 20 weeks—that is also able to bind human disease samples, but importantly does not react to unphosphorylated physiological Tau protein. Antibody can enter the brain and bind both soluble and intraneuronal pTau. Vaccination of mice reduces brain NFT, pathology, indicators of neuroinflammation and improves cognitive function in two different models of memory.

## Introduction

Alzheimer’s disease (AD), the most common type of dementia, is pathologically characterized by the aggregation of amyloid beta (Aβ) plaques, intraneuronal accumulation of hyperphosphorylated and pathological forms of microtubule-associated protein tau (MAPT; pTau) as neurofibrillary tangles (NFTs), synapse loss, and widespread neuroinflammation.^1^ Recent reports have correlated both hippocampal atrophy^2^ and cognitive decline directly to tau pathology in AD.^3–5^ Furthermore, both positron emission tomography (PET), using the tau tracer, ^18^F-AV-1451, and post-mortem neuropathological analysis have provided a strong link between a decrease in mini-mental state exam (MMSE) cognitive scores and NFT burden.^6,7^ Taken together, these studies justify the development of a pTau-targeted therapy to reduce dementia and cognitive decline in tauopathies.

Over the last decade, and in large part due to the disappointing outcomes of many Aβ-specific passive and active immunotherapies,^8^ several groups have begun to investigate various strategies targeting pTau in multiple animal models of AD (reviewed in ref. ^9^). It is evident from these studies that immunotherapies targeting hyperphosphorylated tau^10–13^ appear to be relatively safe and effective in reducing pTau burden. A few of these compounds have recently moved into the clinical trial realm, for example, 8E12 (AbbVie/C2N Diagnostics LLC.), RO7105705 (Genentech Inc.,) and BIIB092 (Biogen) are all monoclonal antibodies (mAbs) that target pTau in passive immunotherapies.^14^

Despite the promising results from efficacy and safety trials of passive immunotherapies, there are several limitations, which undermine their therapeutic value. First, purified mAbs are expensive to produce and often require repeated administrations.^8^ Importantly, affordability is a major concern for developing therapies for Alzheimer’s disease. Particularly, if repeated long-term administration is required to a large AD patient population (~50 million people worldwide) it would simply not be cost-effective. Second, central nervous system (CNS) penetrance and availability of purified mAbs can be hundreds of folds lower compared with those in the serum.^15,16^ Two possible unexpected consequences in a long-term treatment paradigm are the peripheral immunoglobulin (Ig) surge, including vasogenic edema,^17,18^ or on the other hand an extremely slow rate of absorption, depending on the route of administration.^19^ Finally, humanized mAbs can sometimes elicit harmful anti-mAb immune responses, which can impact the effectiveness of therapy.^20^

Active immunotherapy is an alternative approach to overcome these challenges, but the side effects, particularly when using pro-inflammatory adjuvants, which are required to elicit a strong antibody response against self-proteins, can be an even greater safety concern.^21^ Since the immune system is relatively weak in patients who are elderly and affected by AD and related dementias^22^ the induction of antibodies against pTau could be further limited to the already relatively weak immune responses to self-antigens, typical with conventional vaccine approaches.^23^ To address these limitations, we describe an approach that multivalently displays a small, disease-relevant pTau peptide, phosphorylated at threonine 181 (pT181) at high valency on the icosahedral surface of virus-like particles (VLPs) of the RNA bacteriophage Qß.^24^ Vaccination with pT181-Qß induces a rapid and robust antibody response against pTau, that is brain penetrant, and ameliorates neuroinflammation, after only three intramuscular administrations. Immunization with pT181-Qß reduces tau pathology, prevents neuronal cell death, rescues brain atrophy, and preserves delay-dependent and spatial memory in the rTg4510 mouse model of tauopathy (models the familial form of tauopathy called frontotemporal dementia and parkinsonism linked to chromosome-17—tau type or FTDP-17T, with a single P301L point mutation in the exon 10 of *MAPT*).

## Results

### Immunization of pT181-Qß induces robust anti-pT181 antibody titers in both non-transgenic and rTg4510 mice

**Fig. 1:**
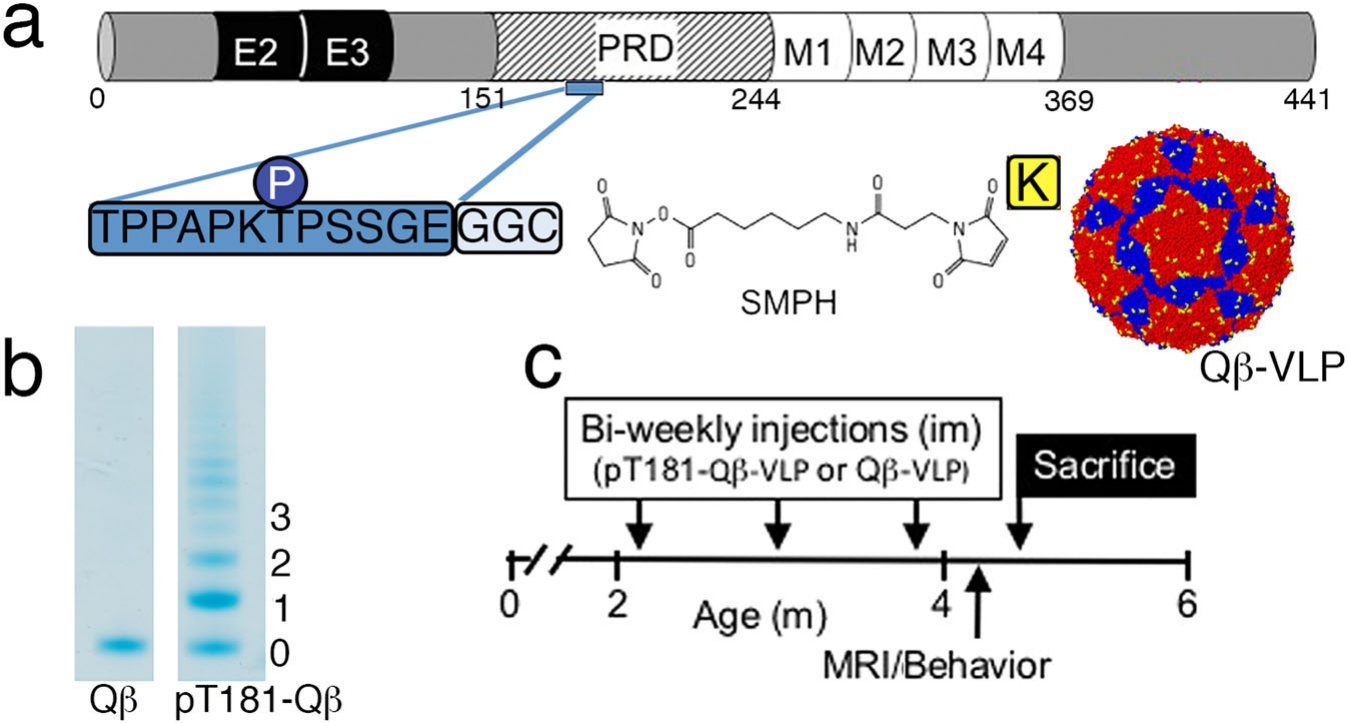
pT181-Qβ conjugation and treatment paradigm. **a** A 13-mer peptide, including phosphorylated epitope Threonine 181 (pT181), was synthesized with two glycines (G) and a cysteine (C) and conjugated to the surface-exposed lysine (K, in yellow) residues on the coat proteins of self-assembled Qß RNA bacteriophage VLPs (AB dimers in red, and CC dimers in blue), using SMPH. **b** An upward mobility shift on a 10% SDS gel indicates the number of peptides conjugated, per bound coat protein. **c** Non-Tg (*n* = 6) and rTg4510 (*n* = 17, separated into two different age- and sex-matched cohorts) were aged to 2 months, received three, bi-weekly intramuscular (im) injections of either: for NonTg: Qß (*n* = 2) or pT181-Qß (*n* = 6); for rTg4510: Qß (*n* = 8) or pT181-Qß (*n* = 9)

A 13-amino acid tau peptide phosphorylated at threonine 181 modified with a two glycine and one cysteine spacer sequence for conjugation (^175^TPPAPKp**T**PPSSGEGGC^190^; referred to as pT181) was selected to test the VLP-based vaccine approach (Fig. 1a). We selected pT181 as a vaccine antigen because it is a cerebrospinal fluid (CSF) biomarker currently used in the diagnosis of AD,^25^ and has been shown to have a better prognostic value in predicting the mortality in patients with AD.^26^ We chose a 16 amino acid sequence because shorter sequences are easier to display on VLPs but are still sufficient to elicit specific antibody responses to a target antigen, thus mimicking a monoclonal antibody-like response. To produce pT181-Qß, the pT181 peptide was conjugated to the surface-exposed lysines (in yellow) on the Qß bacteriophage VLPs using a bifunctional cross-linker (Fig. 1a).^27^ By analyzing the mobility shift of the SDS-PAGE gel we estimate that an average of ~200 pT181 peptides were displayed on the icosahedral surface per Qß-VLP (Fig. 1b). To assess the immunogenicity and effects of the vaccine, 2-month-old non-transgenic (Non-Tg) and tauopathy-prone transgenic rTg4510 mice were administered three biweekly doses of pT181-Qß or Qß control. Following the second dose, serum antibody titers were measured. One-week following the vaccination paradigm, animals were tested in well-validated cognitive tasks, underwent MRI, and were sacrificed for biochemical and neuropathological assessment (Fig. 1c). A separate cohort that underwent the same dosing scheme were processed for flow cytometry 1 week following the final injection. According to previously published studies, deficits in spatial navigation were observed in rTg4510 mice as early as 2.5 months of age^28^ and these deficits are further exacerbated by 4 months of age,^29^ suggesting our approach here is therapeutic.

**Fig. 2:**
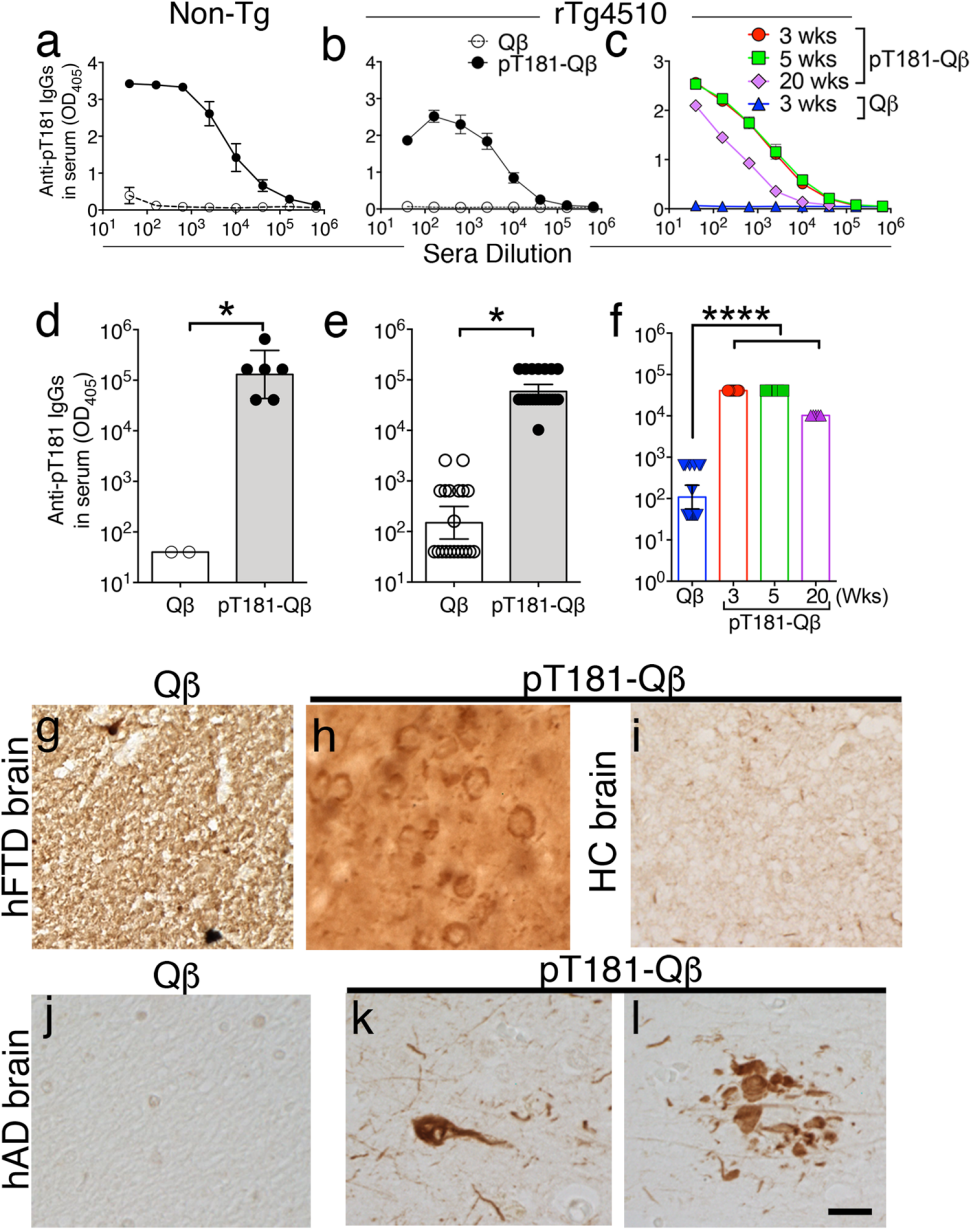
pT181-Qβ vaccination induces a robust and long-lived titer response, and these antibodies can recognize pathological tau in human post-mortem brain tissues. Anti-pT181-IgG titer dilution curves were significantly higher in both pT181-Qß vaccinated Non-Tg **a** and rTg4510 **b** mice vs. Qß controls. Anti-pT181-IgG end-point titer ELISA on sera of Non-Tg **d** and rTg4510 **e** mice was significantly higher in the pT181-Qß- vs. Qß-vaccinated group. Anti-pT181 IgG titer dilution curve **c** and end-point titer **f** remain significantly elevated for at least 4 months post-final immunization in rTg4510 mice (graph displays mean Antibodies derived from the sera of Qß and pT181-Qß-vaccinated Non-Tg mice was used to detect pathology in human frontotemporal dementia (hFTD), healthy control (HC) and human Alzheimer’s disease (hAD) brain sections. Sera derived from Qß-immunized Non-Tg mice was unable to detect any pathology in either hFTD **g** or hAD **j** samples, but sera derived from pT181-Qß-immunized Non-Tg mice was able to detect somatodendritic pTau inclusions in both hFTD **h** and hAD **k** and dystrophic neurites surrounding neuritic plaques in AD **l**, but had no immunoreactivity in HC **i**, scale bar is 20 µm. Graphs **a**–**c** display mean ± SEM, while graphs **d**–**f** display geometric mean ± 95% Confidence Interval, significance values with two groups were determined with a student’s *t*-test, when three groups were analyzed One-Way ANOVA with Dunnett’s multiple comparison's test was used (*p* ≤ 0.05 *, p ≤ 0.0001****). NonTg: Qß (*n* = 2) or pT181-Qß (*n* = 6); for rTg4510: Qß (*n* = 18) or pT181-Qß (*n* = 19); For longevity of the titer response: Qß 3 wks (*n* = 18), pT181-Qß 3 wks (*n* = 9), 5 wks (*n* = 4), 20 wks (*n* = 5)

Owing to its mechanism of action, the repetitively displayed pT181-Qß antigen readily crosslinks B-cell receptors (BCR) and generates a robust immune response via activation of B cells.^30^ To assess this immune response, ELISA was performed on the sera isolated from vaccinated mice, 1 week following the second injection. Robust anti-pT181 IgGs were detected in both Non-Tg (Fig. 2a and d) and rTg4510 mice (Fig. 2b and e). The antibody titers remained elevated even twenty weeks following the final booster (Fig. 2c and f). In contrast, immunization with control Qβ VLPs did not elicit any detectable anti-pT181 antibodies (Abs) beyond what appeared to be naturally occurring anti-pT181 Abs in the rTg4510 mice (Fig. 2a–f). Further, sera derived from pT181-Qβ, but not Qß-, vaccinated Non-Tg mice was able to detect classical somatodendritic pTau^+^ structures in the autopsy brains of human FTD (Fig. 2h), and intracellular pathological inclusions of tau in neurons as well as tau accumulations in dystrophic neurites in the peri-plaque regions in human AD tissue (Fig. 2k–l). Of note, no immunoreactivity was detectable in the brain sections from healthy controls when incubated with sera from pT181-Qß vaccinated Non-Tg (Fig. 2g). Furthermore, sera from Qß-vaccinated Non-Tg mice was unable to detect any pathological structures in human AD brain tissue (Fig. 2j), demonstrating highly specific target-engagement by anti-pT181 IgGs.

**Fig. 3:**
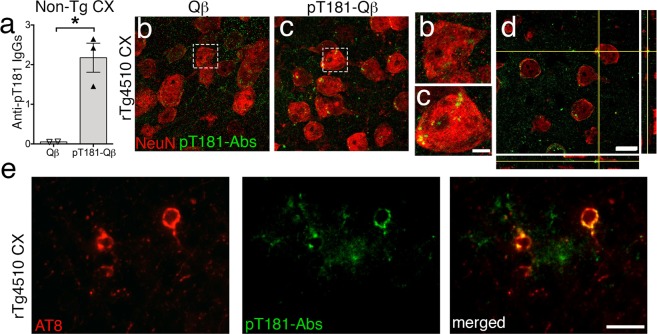
pT181-Qß generated antibodies enter the brain and closely-associate with neurons, positive for pathological tau. Confocal image showing anti-pT181 IgGs were significantly elevated in the cortical lysate of pT181-Qβ vaccinated Non-Tg **a**. The anti-pT181 IgGs, readily detectable in the pT181-Qβ, but not Qβ control, vaccinated rTg4510 brains (**b**, **c**, magnified images of **b** and **c**; Streptavidin-conjugated Alexa Fluro 488 detects pT181 antibodies, in green; NeuN, neurons, in red). Orthogonal images, obtained from the confocal microscope, from immunized mice revealed that pT181 antibodies did not colocalize with NeuN, but were likely in the neuronal cytoplasm, as well as in the brain parenchyma **d**. Epifluorescence images showing anti pT181 IgGs also co-localized to neurons containing pathological tau (AT8) **e**; Streptavidin-conjugated Alexa Fluro 488 detects pT181 antibodies, in green; AT8, red). Graph displays geometric mean ± 95% Confidence Interval, significance value was determined with a student’s *t*-test, (*p* ≤ 0.5 *). Scale bars in **b**–**d** are 10 μm; magnified images of **b**, **c** are 2 μm, scale bar in **e** is 20 μm

To determine whether these disease-specific, anti-pT181 antibodies (Abs or IgGs) entered the brain, an ELISA was performed on the detergent-soluble brain lysates from Qß- and pT181-Qβ-vaccinated Non-Tg mice. There was a significant increase in anti-pT181 IgGs in the brains of Non-Tg mice vaccinated with pT181-Qβ compared to Qβ alone (Fig. 3a). Non-Tg brains were used to avoid any background (naturally occurring) anti-pT181 Abs that could be present in the rTg4510 brain. To determine the CNS infiltration of anti-pT181 Abs, 30-micron-thick brain sections were probed with biotinylated-pT181 tau peptide followed by streptavidin-conjugated Alexa Fluor 488 (green). pT181 Abs were observed in both the extracellular space and inside neurons, as indicated by neuronal nuclei marker NeuN (red) in the cortex of pT181-Qβ vaccinated mice (Fig. 3b, c). Orthogonal images of confocal Z-stack revealed minimal to no co-localization between NeuN and pT181 Abs (Fig. 3d), suggesting that anti-pT181 Abs might be located inside the neuronal cytoplasm, not the nucleus, and this corroborates with previous reports.^31,32^ By confocal microscopy, a Z-stack revealed neurons containing both pT181-Abs and pTau (AT8, in red) as well as some neurons with ONLY pT181-Abs and no pTau, hinting at the possibility of an intraneuronal clearance mechanism (Supplementary Movie 1 and Supplementary Movie 2). Furthermore, by epifluorescence, we observed notable co-localization of anti-pT181 Abs (green) with pTau, (AT8 in red) in the cortex of pT181-Qß vaccinated rTg4510 mice (Fig. 3e). Importantly, anti-pT181 Abs were not observed in the midbrain or brain regions other than cortex/hippocampus. Taken together, these data suggest that anti-pT181 antibodies, generated by pT181-Qß are not only long-lived, but that they enter CNS, target pathological species of tau, localize to neurons, and are also specifically enriched in neurons with AT8+ pTau.

### pT181-Qß vaccination reduces both soluble and insoluble species of pTau

**Fig. 4:**
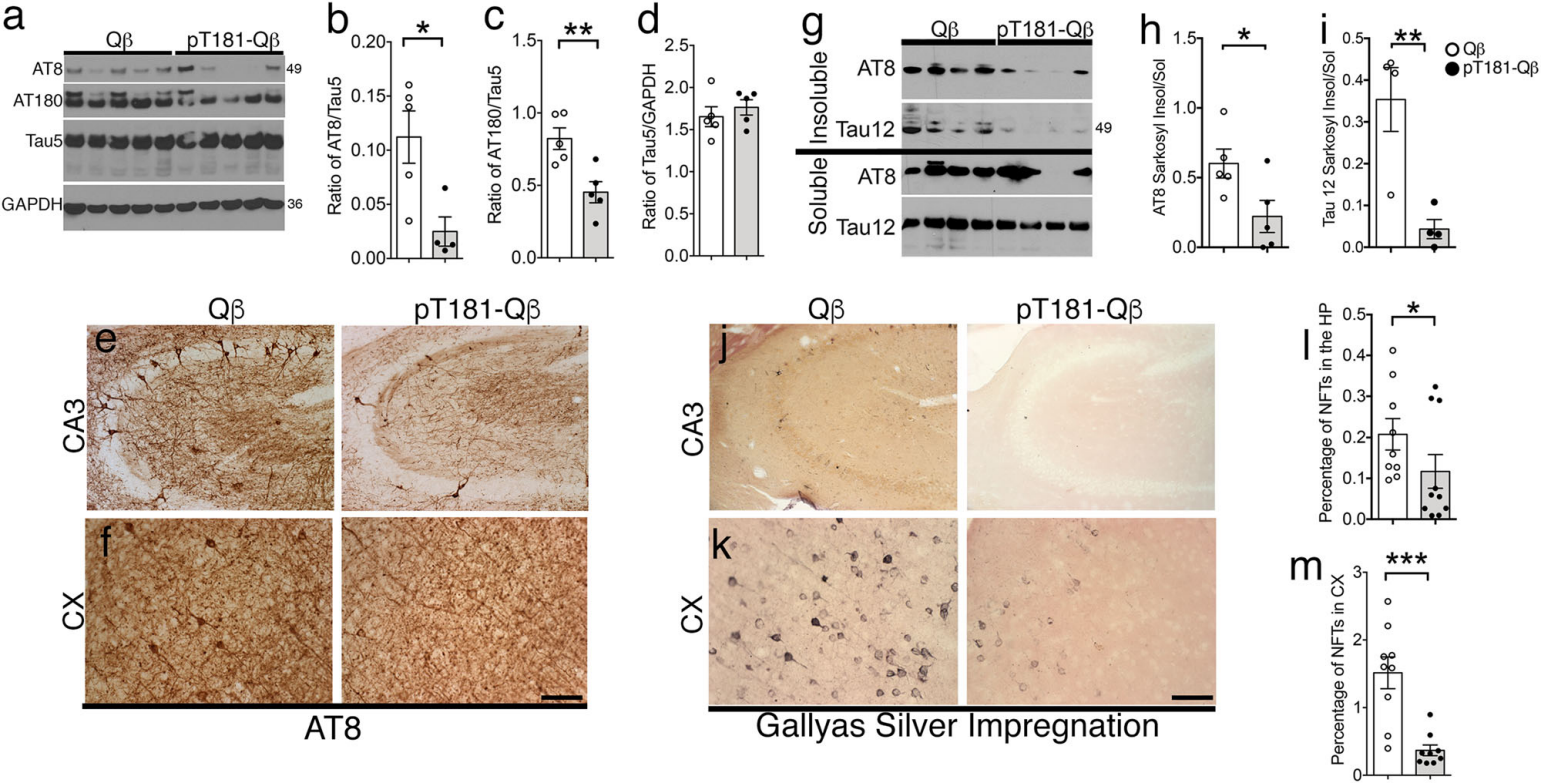
pT181-Qß vaccination significantly reduces soluble and insoluble tau species in rTg4510 mouse model of tauopathy. Western blot of soluble hippocampal lysates **a** reveal that pT181-Qß vaccination, but not Qß, reduces hyperphosphorylated tau species, AT8 and AT180, by 3-fold and 2-fold, respectively **b**, **c**, but does not alter total tau levels **d**, and similarly reduces AT8 immunoreactive neurons in the CA3 region of the hippocampus **e** and the cortex (CX) **f**. Sarkosyl-insoluble fractions from the hippocampus illustrated that pT181-Qß immunization significantly reduced both AT8 and human tau (Tau12), whereas Qß alone had no effect (**g**–**i**). Representative high magnification images of Gallyas silver impregnation revealed qualitative differences in the CA3 **j** and CX **k**. A morphometric quantification illuminated a significant reduction in the number of neurofibrillary tangles (NFTs) present in the HP and CX, (**l**, **m**; and additional information available in Supplementary Fig. 1). All graphs display mean ± SEM, significance values were determined with a student’s *t*-test (*p* ≤ 0.5 *, p ≤ 0.01 **, *p* ≤ 0.001 ***). Scale bar in **e**, **f**, **j**, **k** is 40 μm. Qß (*n* = 5) or pT181-Qß (*n* = 5) rTg4510

Accumulation of hyperphosphorylated tau in the hippocampus and the cortex is a hallmark of tauopathies. In order to determine the efficacy of pT181-Qß vaccination, we immunized rTg4510 mice with either Qß control or pT181-Qß and observed that pT181-Qß immunization significantly reduced soluble hyperphosphorylated tau at both the AT8 (pS202/pS205) and AT180 (pT231) sites in the detergent-soluble hippocampal lysates (Fig. 4a–c), without altering normal, physiological tau (Tau5, Fig. 4d), confirming the specificity of this vaccination against pathological over physiological species of tau. Using immunohistochemistry, we were able to visualize this striking decrease of AT8^+^ neurons in both the hippocampal CA3 and cortex of rTg4510 mice vaccinated with pT181-Qß compared with Qß control (Fig. 4e, f).

When hyperphosphorylated tau becomes insoluble, NFTs are the result, and rTg4510 mice readily accumulate NFTs by 4 months of age, which are detectable by Sarkosyl-insoluble extraction methods in protein lysates and Gallyas silver impregnation in brain tissue. Analysis of AT8 and total human tau (Tau12) in the Sarkosyl-insoluble extracts of hippocampal lysates demonstrated that pT181-Qß vaccination significantly reduced the ratio of insoluble/soluble AT8^+^ pTau and total tau (Tau12) by 2-fold and 3-fold, respectively (Fig. 4g-i). This was further confirmed by Gallyas silver impregnation and quantitative morphometry of the cortex (CX) and hippocampus (HP), where a significant decrease in Gallyas positive NFTs was detected by both the area fraction (percentage of NFTs, Fig. 4j–m), and by absolute number of NFTs present in the CX (Supplementary Fig. 1a, b) and HP (Supplementary Fig. 1c, d). These data demonstrate the forte of pT181-Qß vaccination in reducing not only soluble hyperphosphorylated species of pTau, but also in the targeted degradation of Sarkosyl-insoluble species in the rTg4510 mouse brain.

### pT181-Qß vaccination reduces neuroinflammation and CD3^+^ T-cells

**Fig. 5:**
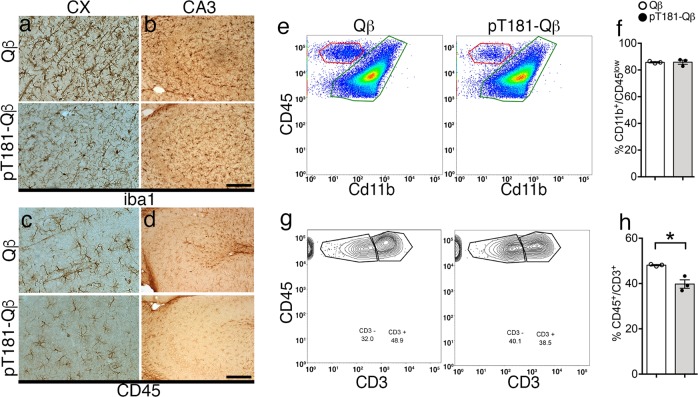
pT181-Qß vaccination attenuates neuroinflammation and reduces CD3+ circulating T cells. Microglia morphology using the pan microglia marker (iba1) was modulated following pT181-Qß vaccination in the CX **a** and CA3 **b**, the phagocytic marker (CD45) was also reduced following pT181-Qß in the CX **c** and CA3 **d**. Isolated microglia from immunized brains (one week following vaccination) revealed that the percentage of resident microglia **e** (CD45^lo^/CD11b^+^, green box) were not affected following immunization (quantified in **f**). However, the presence of T-cells (CD45^hi^/CD3^+^) **g**, from the population of CD45^hi^/CD11b^–^ cells (red box, **e**) was significantly decreased following pT181-Qß immunization **h**. All graphs display mean ± SEM, significance values were determined with a student’s *t*-test (*p* ≤ 0.5 *; Qß (*n* = 4) or pT181-Qß (*n* = 4) rTg4510; scale bar is 20 μm

Neuroinflammation, a key component of Alzheimer’s pathology, is frequently observed to occur alongside and often precede NFT formation, and thus, we hypothesized that pT181-Qß could modulate the inflammatory profile of the brain. To address this, we assayed the levels of message RNA (mRNA) transcripts of inflammatory-related signals in the brain 2 weeks following the final booster. qRT-PCR analysis of the brain did not reveal any alterations in the mRNA profile of pro-inflammatory (*Il1β*, *Tnfα*), anti-inflammatory (*Il10*, *Retnla*) or T-cell-related (*Il18*, *Ifnγ*) gene expression in the rTg4510 mice following immunization (Supplementary Fig. 2a–f). Further investigation revealed that Iba1^+^ microglia were more ramified (less activated) with a notable reduction in CD45^+^ immunoreactivity in both the hippocampus and the cortex of rTg4510 mice vaccinated with pT181-Qß compared to those receiving the Qß control (Fig. 5a–d). Finally, to further characterize the immune cells present in the brain, flow cytometry was performed on isolated mononuclear cells from a separate cohort of pT181-Qß and Qß-vaccinated rTg4510 mice 1 week after the final vaccination (Gating strategy defined in Supplementary Fig. 5). There were no differences in the number of resident microglia (CD11b^+^/CD45^low^, green box) present in the pT181-Qß group compared with Qß-vaccinated rTg4510 mice (Fig. 5e, f). However, there was a significant reduction in circulating T-cells (CD45^+^/CD3^+^) in the rTg4510 mice receiving pT181-Qß vaccination compared with the control group (Fig. 5g, h). It has been well-established previously from ours^33^ and other works,^30^ that small peptides conjugated to Qß-VLPs likely promote Th2-skewed immune responses. Together these data suggest that there are no pro-inflammatory consequences of pT181-Qß vaccination in rTg4510 mice; rather, vaccination with pT181-Qß appears to ameliorate microglial activation and reduce circulating CD3^+^ T-cells in the brains of rTg4510 mice. However, it is important to note that diminished neuroinflammation may simply be a beneficial consequence of the reduction in NFT burden.

### pT181-Qß vaccination ameliorates hippocampal and white matter atrophy

**Fig. 6:**
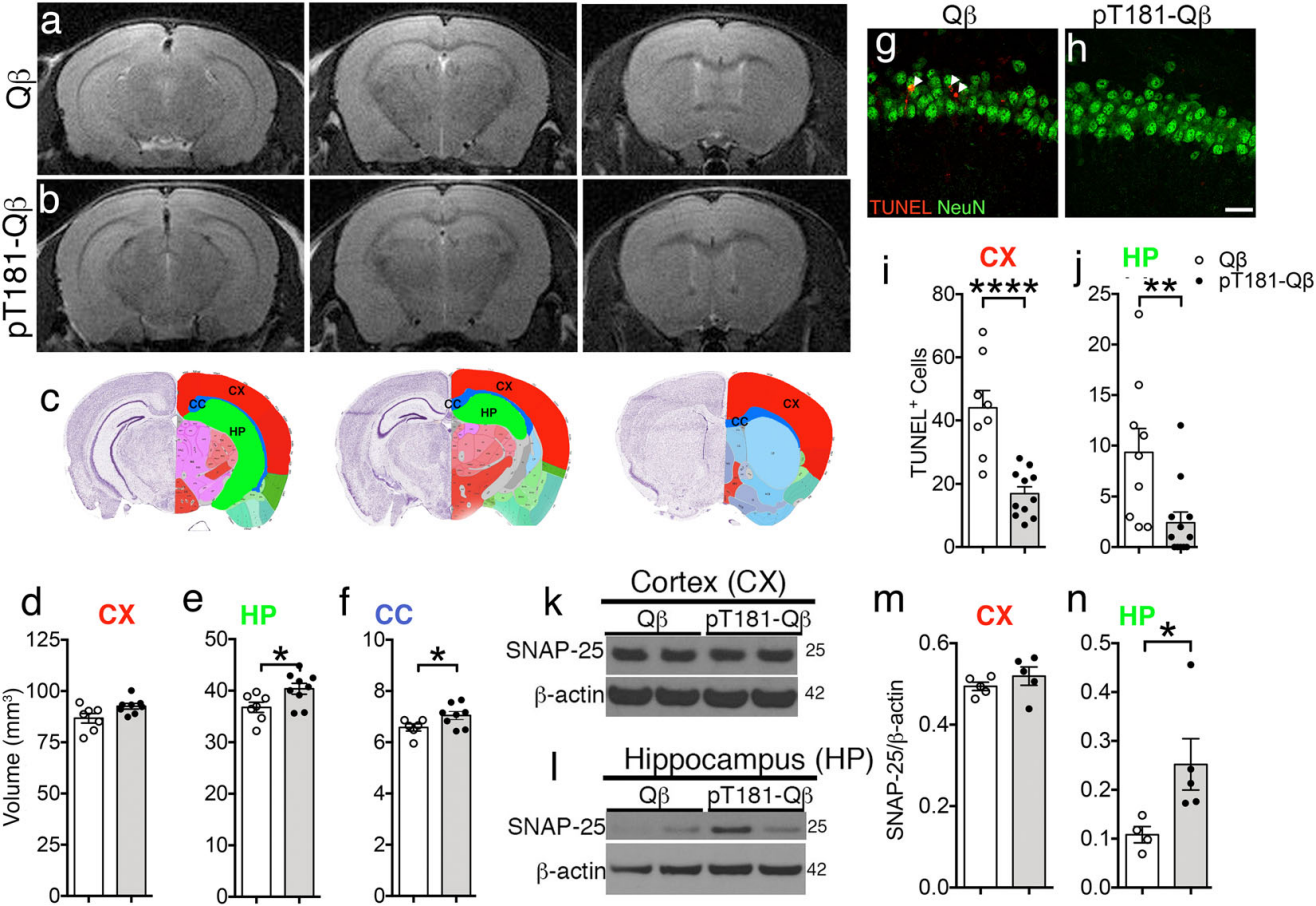
pT181-Qβ vaccination prevents cortical and hippocampal atrophy through amelioration of cell death. T2-weighted MRI spectroscopy of the whole brain (three representative coronal slices displayed matched between groups) revealed that there was significant brain atrophy in the Qß treated rTg4510 **a** which was rescued with pT181-Qß immunotherapy **b**. Allen Brain Atlas was used to draw region of interest (ROI) around the cortex (CX, red), hippocampus (HP, green) and corpus callosum (CC, blue) and VIVOquant was used to analyze the volume of each of these regions in the rTg4510 receiving Qß (white) or pT181-Qß (black) vaccination. pT181-Qß vaccination significantly rescued HP and CC atrophy **d**–**f**, but only slightly rescued atrophy in the CX. There was a decrease in apoptotic neurons, detected by TUNEL/NeuN in the CX and HP (**g**, **h**, quantified in **i**, **j**, respectively) after pT181-Qß vaccination. Western blot of cortical **k** and hippocampal **l** lysates shows unaltered SNAP-25 levels in CX lysates (**k, l**), but there was a significant rescue of SNAP-25 protein in HP lysates (**l**, **n**). All graphs display mean ± SEM, significance values with two groups were determined with a student’s *t*-test (*p* ≤ 0.05 *, p ≤ 0.01 **, *p* ≤ 0.001****). For MRI on rTg4510: Qß (*n* = 8) or pT181-Qß (*n* = 9), for TUNEL/NeuN double immunofluorescence, Qß (*n* = 6) or pT181-Qß (*n* = 6), for western blot Qß (*n* = 5) or pT181-Qß (*n* = 5); scale bar is 20 μm

Neuronal cell loss and brain atrophy are common pathological hallmarks in many neurodegenerative diseases, including AD, and to this point we utilized small animal T2-weighted MRI (T2_w_) to determine whether the reduction in pTau and amelioration of neuroinflammation could affect the structural integrity of the brain. Thus, hand-drawn regions of interest (ROI), using the Allen Brain Atlas, were applied to each slice of an magnetic resonance (MR) scan to assess the effects of pT181-Qß vaccination on the volume of the cortex (CX, red), hippocampal formation (HP, including entorhinal cortex, green), and corpus callosum (CC, blue) (Fig. 6a–c). T2_w_ MRI volumetric analysis revealed that rTg4510 mice vaccinated with Qß alone (Fig. 6a), experienced significant brain atrophy compared with the structural stabilization seen in the HP and CC, specifically, following pT181-Qß (Fig. 6b) vaccination (Fig. 6c–f). We hypothesized that this reduction in hippocampal atrophy in the pT181-Qβ vaccinated rTg4510 mice was due to vaccine-induced neuroprotection in this brain region. Accordingly, we used TUNEL combined with NeuN (a neuronal marker) to assess neuronal apoptosis and observed a three-fold decrease in apoptotic neurons in the hippocampus and cortex of rTg4510 mice vaccinated with pT181-Qβ compared with Qβ vaccinated controls (Fig. 6g–j). Finally, pT181-Qβ vaccination prevented synaptic loss, and this was demonstrated by western blot of the higher the synaptic protein—SNAP25, which was elevated, only in the hippocampus, but not the cortex of pT181-Qß vaccinated rTg4510 compared to Qβ controls (Fig. 6k–n). These data suggest that vaccination, which effectively reduces the burden of both soluble and insoluble aggregates of pTau, also decreases apoptotic neurons, which translates into amelioration of both gray and white matter loss in the hippocampus and corpus callosum, respectively, and prevents loss of SNAP25. Notably, there were no additional white matter structural deficiencies in the rTg4510 mice at 4 months of age (Supplementary Table 1), which corroborates with previous reports that structural deficiencies do not occur in the rTg4510 mice until after 5.5 months of age.^34^

### pT181-Qβ vaccination improves recognition and spatial memory in rTg4510 mice

**Fig. 7:**
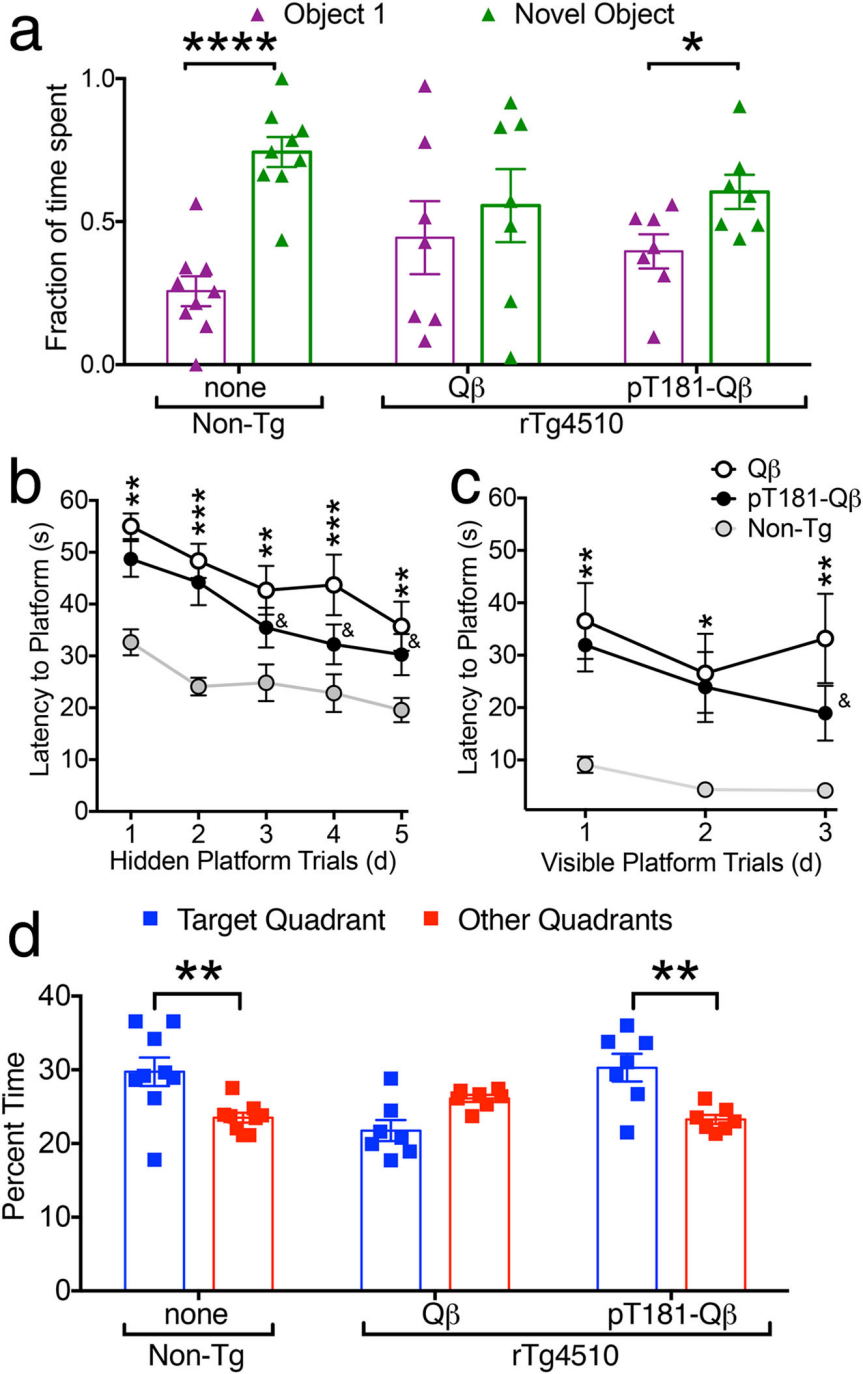
pT181-Qβ vaccination rescues delay-dependent memory and spatial learning and memory. During the novel object recognition task, animals are exposed to one familiar and one novel object in a 75 cm^2^ arena, with an interval of 24 h between the sample phase (two identical objects, Supplementary Fig. 3) and the novel phase. Of course, non-Tg mice spent a larger fraction of time with the novel object over the familiar object, and Qß-vaccinated rTg4510 did not, but this impairment was significantly rescued with pT181-Qß-vaccination in the rTg4510 mice **a**. Hidden platform training trials of the Morris water maze (MWM), revealed Non-Tg (gray) animals learned the task faster than rTg4510, until day 3, when pT181-Qβ vaccinated rTg4510, were not statistically different from the Non-Tg, but latency to platform was significantly faster compared with the Qß-vaccinated rTg4510 **b**. During the visible platform trials, the latency to platform was statistically different between Non-Tg or rTg4510, except for Day 3, when the pT181-Qβ vaccinated rTg4510 out-performed the Qβ-treated rTg4510 and were not different than Non-Tg **c**. During the probe trial (platform removed) **d**, Non-Tg mice spent a larger percentage of time in the target quadrant compared to the other quadrants where the Qß-treated rTg4510 did not show a preference, but pT181-Qß vaccination rescued this deficit in the rTg4510. All graphs display mean ± SEM, Two-Way ANOVA with Sidak’s multiple comparison’s test for Novel object-familiar object for each group **a**, Two-Way ANOVA with Tukey’s multiple comparisons test (learning curve, **b**, **c**), or Sidak’s multiple comparison’s test for Target Quadrant-other quadrant for each group (MWM probe trial, **d**), *p* ≤ 0.05 *, *p* ≤ 0.01 **, *p* ≤ 0.005 ***, *p* ≤ 0.0001 ****, ^&^ = not significant compared to Non-Tg. Data represent two separate cohorts of mice run at two different experimental time points, there were no significant differences detected between experiments and all experiments were counter-balanced to include all groups. Non-Tg *n* = 12, Qß-rTg4510 *n* = 9, pT181-Qß-rTg4510 *n* = 10

We next tested if this pT181-Qβ vaccination has any effect on the cognitive function in the rTg4510 mouse model of tauopathy. To that end, we assessed 4-month old untreated Non-Tg, and Qß- and pT181-Qß vaccinated rTg4510 mice through two well-characterized behavioral assessments, novel object recognition (NOR) and Morris water maze (MWM) tasks, which measure delay-dependent and spatial memory, respectively. Whereas none of the experimental groups preferred either of two identical objects in the acclimation trial of the NOR task (Supplementary Fig. 3a), non-Tg mice significantly preferred the novel object to the familiar object, as expected (Fig. 7a). Qß-vaccinated rTg4510 mice were unable to distinguish between the familiar and novel objects, but pT181-Qß vaccinated rTg4510 mice significantly preferred the novel object to the familiar one (Fig. 7a). Importantly, there were no significant differences in distance traveled (cm) or velocity (cm/s) across acclimation, sample or test days in any of the groups or treatments (Supplementary Fig. 3b). Together, these data indicate that pT181-Qß-vaccination rescues delay-dependent recognition memory in the rTg4510 mice.

We next used the MWM task to assess whether vaccination also has a positive effect on hippocampal-dependent spatial learning and memory. By day 3 of hidden platform trials, pT181-Qβ-vaccinated rTg4510 mice found the submerged platform significantly faster than Qβ-vaccinated controls, but not as quickly as non-Tg mice (Fig. 7b). We also observed a trend toward enhanced spatial memory and learned behavior in pT181-Qß-vaccinated rTg4510 mice in the visible platform trials (Fig. 7c). Finally, we also measured spatial memory by removing the platform from the tank and then measuring the percentage of time mice spent in each quadrant of the maze. Unlike Qß-vaccinated rTg4510 mice, both untreated Non-Tg and pT181-Qß-vaccinated mice spent significantly more time in the target quadrant compared with the other quadrants (Fig. 7d). The observed behavioral differences were not due to impaired mobility, as only small non-significant differences in path length (cm) and velocity (cm/s) between the rTg4510 mice were seen, regardless of treatment (Supplementary Fig. 3c, d). Importantly, rTg4510 were significantly more hyper-active compared with Non-Tg mice on days 2–5 of the hidden trials of MWM, and vaccination did not seem to alter this phenotype. Taken together, these data suggest that vaccination with pT181-Qß can rescue both spatial memory and improve hippocampal-dependent learning in rTg4510 mice.

## Discussion

This study illustrates the effectiveness of a Qß-VLP-based vaccine targeting a pathologically relevant species of tau (pT181) in one of the most pathologically aggressive mouse models of tauopathy (rTg4510 mice). First, immunization with pT181-Qß elicits a robust antibody titer against pTau, these Abs are long-lived, they specifically target pTau, readily enter the brain, and pathological tau inside neurons. Most importantly, the pT181-Qß vaccine reduces levels of hyperphosphorylated soluble pTau and insoluble NFTs, dampens neuroinflammation, and prevents hippocampal atrophy. Reduction in pathological consequences following this immunotherapy translated into improved cognitive function using well-established measurements of delay-dependent and spatial learning and memory tests. Therefore, we believe that this report provides compelling evidence that a VLP-based immunotherapy approach can be beneficial in preventing tau pathology and could be applicable to diverse human tauopathies, such as Alzheimer’s disease (AD), frontotemporal dementia (FTD), traumatic brain injury (TBI), and chronic traumatic encephalopathy (CTE).

Other active immunotherapy strategies targeting pTau have utilized tau peptides, and some have shown success in reducing tau pathology and rescuing cognition in mouse models of tauopathy. In one example, pTau peptide (MAPT_379–408_, pS396/404) formulated with an adjuvant (Adju-Phos), induced tau-reactive antibodies, reduced MC1 and PHF1 positive neurons in the brains of TgP301L mice and rescued cognitive function.^10^ However, the ability of this therapy to decrease Sarkosyl-insoluble tau species, reduce NFTs, or modulate neuroinflammation was not assessed. Two active immunotherapies targeting pTau that rely on a similar strategy are now in Phase I clinical trials: AADvac1, a tau peptide 294–305 linked to keyhole limpet hemocyanin (KLH) and administered with an alum adjuvant,^35^ and ACI-35, a 16-amino acid tetra-palmitoylated phosphorylated tau peptide (encompassing the pS396/pS404 pTau residue) with a monophosphoryl lipid A adjuvant (MPLA).^36,37^ Preliminary results from these trials indicate that AADvac1 is safe and immunogenic.^38^ Nevertheless, based on our results, we believe that the VLP-based strategy presented here, is safer and more immunogenic than a KLH-conjugate or a peptide-adjuvant-based active immunotherapy, because the VLP-approach does not require any additional adjuvant.^39^ Peptide-based vaccines do elicit antibody responses; however, they are short-lived and weaker than the highly multivalent presentation of antigens (like pT181-Qß), and this can facilitate better crosslinking of B cell receptors.^40^ Anti-pT181 antibody levels with pT181-Qß vaccine were several orders of magnitude higher and long-lasting compared to those with conventional adjuvants. An important point to consider with this therapy, is the potential downside that once an immune response is initiated, that there could be an inability to halt or restrain this response. However, we feel that since the pT181-Qß avoids cytotoxic T-cell involvement, as previously published^30,33,41^ and clinical evidence from a study in 24 patients, using Qß-VLP reported only mild irritation at injection site,^42^ we believe that inflammatory responses due to vaccination might be very minimal.

Interestingly, a vaccine comprised of an Aß peptide conjugated to Qß-VLP was shown to reduce pathology and to rescue cognition in an animal model of Alzheimer’s disease^43^ and has progressed into Phase III clinical trials (CAD106). Thus far, it has shown a favorable safety profile and is highly immunogenic,^44^ but whether or not it decreases pathology or, more importantly, ameliorates cognitive decline in humans has yet to be reported. Recent work, investigating pathological tau (tau_294–305_) conjugated to a Hepatitis B-core VLP was shown to induce immunity against tau, reduce truncated tau and rescue behavior in a P301S mouse model of tauopathy.^45^ However, since many modifications in tau (including phosphorylation, truncation, oligomerization, etc.) have been demonstrated to contribute to disease pathogenesis, the most effective epitope of tau for immunological targeting is still debatable. While an epitope in the middle of the microtubule-binding region of tau (which is known to promote tau aggregation)^46^ was used in this hepatitis B-core VLP tau_294–305_ study, such an approach has the potential to compromise normal microtubule-binding functions of tau. For example, in that study they reported reduced levels of normal physiological tau (Tau5).^45^ In contrast, in the report presented here, pT181-Qß did not reduce total soluble non-pathogenic tau species (Fig. 4d) but instead specifically reduced hyperphosphorylated tau and the ratio of insoluble/soluble tau.

Vaccination with pT181-Qß generated a high-titer response against pTau, but it remains unclear how these antibodies function in sequestering and/or degrading pTau. A recent study has demonstrated that immunotherapy-generated antibodies enter the brain through Fc receptors, form complexes with antigens and these antibody-antigen complexes are cleared through peripheral sink mechanisms,^47^ but we did not detect blood-brain barrier disruption via Hematoxylin and Eosin, so this mechanism is unlikely. While another report, using fluorescently labeled anti-pTau IgGs on a slice culture model of pTau in mouse brain, have observed antibodies to co-localize with phosphorylated tau inside the neurons of tau transgenic mice.^10^ Further, Collin et al. observed neuronal uptake of passive immunotherapy antibodies and co-localization with lysosomes inside neurons suggesting a potential intracellular clearance mechanism, as well.^48^ In contrast, Yanamandra and colleagues, have suggested that anti-tau antibodies can inhibit tau seeding and spreading through extracellular sequestration.^49^ Our data seems to support that pT181-Qß-generated antibodies could be functioning through both extracellular and intracellular clearance mechanisms.

Another possible advantage of the VLP approach is the lack of neuroinflammation, which was evident in our study, mainly because neuroinflammation exacerbates tau pathology and worsens cognitive function as we have previously demonstrated.^50–52^ This underlines the importance of developing an immunotherapy strategy that avoids pro-inflammatory consequences. Some immunization studies targeting pTau failed to characterize the neuroinflammatory profile,^10,53^ while another study reported detrimental elevated neuroinflammation following immunization with a phospho-tau peptide using an adjuvant cocktail: complete-Freund's adjuvant (CFA) and pertussis toxin (PT).^54^ One study that utilized semi-quantitative immunohistochemistry approach did not observe any differences in their treated group,^12^ while the Hepatitis B-Core VLP tau_294–305_ approach only reported a reduction in pan-microglial marker, iba1, in P301S mice, without elaborating on further neuroinflammatory consequences.^45^ While the specific mechanisms for the attenuation of brain inflammation in the present study remain unclear, one possibility is that the reduction of tau pathology may itself subdue microglial activation, since the two are intimately linked. Alternatively, since B cells are the main immune cells responsible in driving the immune response when using the VLP platform,^30^ the therapy itself may promote a Th2-skewed response, as previously reported.^55^ Furthermore, previous work from our group did not detect any proinflammatory T-cell response after immunization with an Aß-Qß VLP.^27^ Nevertheless, our study shows a clear reduction in microglial activation, with no evidence of concomitant pro-inflammatory Th1 cell proliferation/activation, which has been negatively associated with clinical outcomes in a past Aβ-targeted immunotherapy.^56^

One unique aspect of this work is that, a small animal MRI has been used to investigate the effectiveness of VLP-based pTau immunotherapy on the prevention of brain atrophy. We believe that such metrics will have potential translational implications for our vaccine approach in the near future. A clear decrease of neuronal cell loss and amelioration of hippocampal atrophy suggests that clearance of pTau/NFTs is neuroprotective. The lack of white matter response in the corpus callosum is not surprising given that white matter changes in diffusion tensor imaging paradigms like Fractional Anisotropy or Mean Diffusivity are not typically observed until after 5.5 months of age in rTg4510 mouse model of tauopathy.^34,57,58^ Therefore, a later time point in this strain or vaccination in another tauopathy strain with known white matter deficits could more accurately address how pT181-Qß immunotherapy may affect axonal injury.

We recognize that there may be a few limitations with the VLP-based vaccine approach against tauopathies. For example, if there are unexpected side effects in human trials (which is unlikely as two of the VLP-based vaccines are already FDA approved for human papilloma virus—Gardasil® and Hepatitis B—Recombivax HB^®^), it would take longer to stop the pT181-Qß induced adaptive immune response. To address this limitation, additional emphasis on the careful analysis of inflammation, specifically in human subjects, may be warranted. Another limitation is that reduction of pathological tau may be insufficient to completely alter the course of the disease. This could require additional intervention or combinatorial therapy beside VLP-based vaccination against tauopathies. Nevertheless, these encouraging data demonstrate target-engagement of pathological tau, in a VLP-based approach and warrants further investigation for clinical development. In conclusion, pT181-Qß may represent a promising new potential therapy against pTau burden in tauopathies.

## Methods

### Ethical statement

The University of New Mexico (UNM) Institutional Animal Care and Use Committee (IACUC) approved (IACUC protocol #s: 19-200841-B-HSC (Breeding); 18-200761-HSC (Experimental)) all animal procedures described in the study.

### Human brain tissue samples

Human frontotemporal dementia (hFTD) brain tissue samples were kindly provided by Northwestern Cognitive Neurology & Alzheimer’s Disease Center (CNADC) Neuropathology Core. Paraffin-embedded human healthy control (HC) and human Alzheimer’s disease (AD) samples were obtained from the Neuropathologist in house at UNM and through the Office of Medical Investigator (OMI). The University of New Mexico and the Institutional Review Board approved the use of all human autopsy specimens under exempt status.

### Production of pT181-displaying Qβ-VLPs

Qβ-virus-like particles (Qβ-VLPs or Qβ) were produced in *Escherichia coli* (*E. coli*) using methods previously described.^59,60^ The target antigen, microtubule-associated protein tau (pathological tau or pTau) peptide phosphorylated at threonine 181 (pT181) was synthesized (American Peptide, USA) and modified to include a C-terminal cysteine residue preceded by a 2-glycine-spacer sequence, underlined (Fig. 1a, ^175^TPPAPKp**T**PPSSGEGGC^190^). pT181 peptide was conjugated to surface-exposed lysines (K, pictured in yellow, Fig. 1a) on Qβ-VLPs using the bifunctional cross-linker succinimidyl 6-[(beta-maleimidopropionamido) hexanoate] (SMPH; Thermo Fisher Scientific, catalog # 22363) (Fig. 1a).^27,41^ Qß-VLP molecular model drawn with Jmol: an open-source Java viewer for chemical structures in 3D, http://www.jmol.org/. Efficiency of conjugation was confirmed via gel electrophoresis on a 10% SDS denaturing polyacrylamide gel (Fig. 1b, uncut gel in Supplementary Fig. 4).

### Animals and treatment

Bi-transgenic rTg4510 were created by crossing the tet-transactivator line: Tg(Camk2a-tTA)1Mmay (JAX, stock# 007004) to the Tet responsive element line: Tg(tet0-MAPT*P301L)#Kha/J (JAX, stock# 015815)^28,61^ and C57Bl6/J mice (Non-tg, JAX, stock#000664) were all obtained from the Jackson Laboratory. Animals were housed in a specific pathogen free (SPF) facility, in a 12 h light/dark cycle with *ad libitum* access to food and water, in 85 in^2^ ventilated microisolator cages, supplemented with sterilized and autoclaved TEK fresh standard crinkle bedding; environmental enrichment included tissue paper, wooden twigs, and an elevated penthouse insert. Mice used in this study were not used for breeding and were housed by sex at a density of two to five mice per cage.

All animals chosen for experimental manipulation were healthy, of average weight, and had no history of any medical conditions including rectal prolapse, skin dermatitis, or malocclusion. 2-month-old Non-Tg and rTg4510 mice, were treated with three, bi-weekly intramuscular (im) injections into the rear hind-limb (Fig. 1c). They received either: unconjugated control vaccination Qß-VLP or Qß-VLP conjugated to a pTau peptide (pT181-Qß) at an approximate concentration of 5 μg/injection into the rear hind paw. No adverse events were observed in either Qß or pT181-Qß group regardless of age, genotype or sex.

Following the treatment, cohorts of mice were run through a battery of cognitive assessments (NOR and MWM, see below), and then imaged using a 4.7 Tesla Magnetic Resonance Imager (MRI) prior to sacrifice (Fig. 1c). Following behavior and imaging, mice were deeply anesthetized with Avertin and transcardially perfused with phosphate buffer. The brains were removed for biochemical and neuropathological analysis following perfusion. The left hemisphere was immersion fixed in 4% PFA (paraformaldehyde, Electron Microscopy Services, catalog # RT15713) for neuropathological analysis via immunohistochemistry, and the hippocampi from the right hemispheres were micro-dissected, wet weights were recorded, snap frozen in liquid nitrogen and stored at −80 C until subsequent biochemical analyses. The rest of the brain fractions were also weighed and snap frozen in liquid nitrogen for gene expression analysis.

### Characterization of antibody response in serum

To assess peripheral antibody titer, blood plasma was collected prior to the final immunization via retro orbital capillary collection and analyzed via ELISA (enzyme-linked immunosorbent assay). To assess the length of peripheral antibody titer over time, blood plasma was collected at the time of sacrifice and for one cohort, 4 months post injection. Anti-pT181 specific IgG titer was determined by end-point dilution ELISA, using pT181 16-mer peptide as the antigen. Briefly, Immulon-2 plates were incubated with 500 ng streptavidin (Thermo Fisher Scientific, catalog #434301) in pH 7.4 phosphate-buffered saline (PBS) overnight (ON) at 4 °C. Following washing, SMPH (Thermo Fisher Scientific) was added to wells at 1 μg/well and incubated for 2 h at room temperature (RT). pT181 was added to the wells at 1 μg/well and incubated overnight at 4 °C. The plate was subsequently blocked with 0.5% milk in PBS for 2 h. Four-fold dilutions of plasma were added to each well and incubated for 2.5 h. The wells were probed with horseradish peroxidase (HRP)-conjugated secondary antibody [goat anti-mouse-IgG (Jackson ImmunoResearch, catalog #115-005-003; 1:4000)] for 1 h. The reaction was developed using 3,3′,5,5′-tetramethylbenzidine (TMB) substrate (Thermo Fisher Scientific, catlog # 34028) and stopped using 1% HCl. Reactivity of sera for the target antigen was determined by measuring optical density at 450 nm (OD_450_). Wells with twice the OD_450_ of the background were considered to be positive and the highest dilution, with a positive value, was considered the end-point dilution titer.

### Characterization of antibody response in the cortical lysate

To assess blood-brain barrier (BBB) penetrance of the generated antibodies into the CNS, cortical brain tissue was removed from perfused mice and homogenized according to the Western Blot methods (details below) and supernatants were analyzed via ELISA. Procedures followed were as indicated above; except cortical brain homogenates were diluted only 10-fold into corresponding wells. Reactivity of the cortical brain lysate for the target antigen was determined by measuring optical density at 450 nm (OD_450_). Wells with twice the OD_450_ of the background, were considered to be positive.

### Identification of the spatial location of anti-pT181 antibodies in the brain

To detect the presence of anti-pT181 IgGs in the brain tissue, free-floating 30 μm thick hemi brain sections derived from untreated Non-Tg, and Qß- and pT181-Qß-immunized rTg4510 mice were carefully processed for both double immunofluorescence and confocal microscopy with a 3-day protocol. First tissue was incubated with a biotinylated pT181 peptide (synthesized by American Peptide, to detect the anti-pT181 IgG antibodies present in the brain, diluted at 1:100) for 24 h (hrs). Followed by a subsequent 1 h incubation with a Alexa Fluor488-conjugated streptavidin secondary antibody (JAX Immunoresearch, Cat #016–540-084, diluted at 1:1000). Neurons, or pathological tau, were detected with an overnight incubation with NeuN (neuronal nuclei, Millipore, MAB 377, diluted at 1:100), or AT8 (pTau corresponding to pS202/pThr205, Thermo Fisher Scientific, MN1020, diluted at 1:500), respectively. Neurons, or pathological tau, were visualized with an anti-mouse Alexa-Fluor® 555-conjugated secondary antibody (Thermo Fisher, A-21422, diluted at 1:1000) for 1 h. Then tissue was affixed to a glass slide, and coverslipped with a hardset-mounting media, DAPI (4′,6-diamidino-2-phenylindole which is a blue-fluorescent-labeled DNA stain) Hardset Reagent (Vector Labs, Cat #H-1500).

### Confirming the specificity of anti-tau IgG in the sera of immunized Non-Tg mice

Pooled sera from Qß-immunized (*N* = 2) and pT181-Qß immunized (*N* = 6) Non-Tg mice were diluted 1:500 in standard blocking solution (5% goat sera/0.4% PBS-Triton X) and incubated with brain tissue from a patient diagnosed with fronto-temporal dementia overnight at 4 °C. Anti-IgG antibodies were detected with biotinylated-goat anti-mouse secondary for 1 h at room temperature, followed by a subsequent incubation with Avidin/Biotin enzyme Complex (ABC reagent, Vector Labs, catalog # PK-6100,) for 1 h at room temperature. Immunoreactive signals were developed by incubating sections in 3-3′-diaminobenzadine (DAB) reagent (Vector Labs, catalog #SK-4100). In a separate experiment, paraffin-embedded tissues from 1 healthy control subject (80 yrs old), and 1 diagnosed AD (88 yrs old) were first deparaffinized through warming on a hot plate at 55 °C for 30 min, and sequential incubations in xylene, re-hydration with subsequently more dilute concentrations of ethanol: 100, 95, 70% and then water, and blocked with standard blocking solution (5% goat sera/0.4% PBS-Triton X) for 1 h. Tissue was then incubated with pooled sera from Qß-immunized (*n* = 2) and pT181-Qß immunized (*n* = 6) Non-Tg mice were diluted 1:500 in standard blocking solution overnight at 4 °C. Anti-IgG antibodies were detected with biotinylated-goat anti-mouse secondary for 1 h at room temperature, followed by a subsequent incubation with Avidin/Biotin enzyme Complex (ABC reagent, Vector Labs) for 1 h at room temperature. Immunoreactive signals were developed by incubating sections in 3-3′-diaminobenzadine (DAB) reagent (Vector Labs). At the conclusion of both the fresh frozen and paraffin-embedded samples, samples were dehydrated, cleared with Xylene, and coverslipped with Permount.

### Immunohistochemistry

Free-floating sections (30 µm thick) derived from multiple mouse brains per group were utilized for all of the immunohistochemical and immunofluorescence analysis. The antibody dilutions were: NeuN (Millipore, MAB377) at 1:100, CD45 (BioRad, MCA43R) at 1:250; AT8 (Thermo Fisher Scientific, MN1020) and Iba1 (WAKO, 019-19741) at 1:500 incubated overnight at 4 °C. Secondary antibodies conjugated to either Alexa Fluor® dyes (1:1000, for immunofluorescence from Thermo Fisher) or biotin (1:250, for immunohistochemistry from Jackson Immunoresearch) were used. Sections were then either mounted with DAPI Hardset Reagent (Vector Laboratories; for immunofluorescence) or incubated with Avidin/Biotin enzyme Complex (ABC reagent, Vector Laboratories; for immunohistochemistry) reagent for 1 h at room temperature. Immunoreactive signals were developed by incubating sections in 3-3′-diaminobenzadine (DAB) reagent (Vector Laboratories). Slides were serially dehydrated, cleared with xylene and mounted with permount (Thermo Fisher).

For double labeling with TUNEL (Terminal deoxynucleotidyl transferase dUTP nick end labeling, TMR red, Millipore, Cat # 12156792910) and NeuN, first the 30 μm sections were incubated with 2 N HCl for 30 min at 37 °C to allow for nuclear permeabilization followed by neutralization with 0.1 M sodium borate buffer (pH 8.6) for 10 min at room temperature. After washing multiple times with PBS, the sections were processed for antigen retrieval as described above. Then the sections were blocked in blocking buffer and processed for TUNEL staining as per manufacturer’s instructions (Millipore) with a one-hour incubation in a humidified incubator. After washing several times, the sections were processed for immunofluorescence with a mouse monoclonal antibody against NeuN overnight, followed by a 1 h incubation with Alexa Fluor® 488 anti-mouse secondary antibody (Thermo Fisher Scientific, catalog # A-11001).

### Gallyas silver impregnation

The Gallyas silver impregnation staining technique was performed on 30 µm free-floating brain sections as previously described, with modifications for free-floating tissue samples.^62,63^ Briefly, 30 μm thick sections were washed with water before being incubated with 5% Periodic acid for 5 min, then washed with water again. Next, tissue sections were briefly incubated with a solution of Alkaline Silver iodide (containing sodium hydroxide, potassium iodide, and silver nitrate) for 1 min. Tissues were then incubated for 10 min in 0.5% glacial acetic acid and then developed in developer solution for a maximum of 5 min. Developer working solution was mixed at a ratio of 3 volumes of II to 10 volumes of I first, prior to the addition of 7 volumes of III: stock solution I: 5% sodium carbonate; stock solution II:0.2% Sodium nitrate, 0.2% silver nitrate, 1% tungostosilicic acid; stock solution III: 0.2% ammonium nitrate, 0.2% silver nitrate, 1% tungostosilicic acid, 73% wt/vol formaldehyde (neat); Then tissues were rinsed briefly with 0.5% glacial acetic acid, rinsed with water, and then incubated with 0.1% gold chloride for 5 min. They were rinsed once more with water, and then incubated with 1% sodium thiosulfate for 5 min. Sections were washed once more with water and then mounted to glass slides. Once dry, they were rinsed carefully with xylene and then glass coverslips were affixed with permount mounting media.

### Microscopic analysis and quantitative morphometry

Bright field and fluorescence images were acquired using an Olympus BX-51 microscope (Olympus America Inc.), equipped with an Optronics digital camera and Picture Frame image capture software (Optronics). This microscope was used in capturing the images of sera from Non-Tg vaccinated with Qß or pT181-Qß on human autopsy tissue, anti-pT181 antibodies present in the brain of pT181-Qß vaccinated mice, the co-localization of pT181 Abs with AT8, AT8 immunoreactivity and Iba1 and CD45 positivity. Confocal images of anti-pT181 antibodies present in the brain of pT181-Qß vaccinated mice were captured via Zeiss LSM510 Meta confocal and were processed via Zen software (Zeiss, Germany) for both maximum Z-projection images (Fig. 3b, c), orthogonal images (Fig. 3d) and Supplementary Movies 1 and 2.

Tiled images of the sagittal plane from Gallyas Silver Impregnation-stained sections were assembled via Photoshop (Adobe, US) and quantified via Fiji/Image J (NIH). Briefly, the cortical and hippocampal regions of all animals were recreated using images obtained at 40X and stitched together in Photoshop (Supplementary Fig. 1). Next, the images were saved and exported as jpegs for analysis in ImageJ software (NIH). The regions of interest (ROI) were drawn around the hippocampus (HP) and cortex (CX) and the thresholding tool was used to discern the Gallyas+ cells (NFTs) from the background. The area fraction was determined as percent positive cell area divided by the area of the region of interest^51^ (*n* = 6/group, 3 separate tiled images per animal and 6 animals per group).

### Western blotting

Hippocampal brain tissue was homogenized in 10 volumes of Tissue Protein Extraction Reagent (TPER®, Thermo Scientific, catalog # 78510) with phosphatase and protease inhibitor cocktails (Sigma Aldrich, P5726 and P8340, respectively), and then sonicated on ice. Soluble hippocampal lysates were centrifuged at 12,000×*g* for 30 min at 4° C and samples were prepared with LDS and RA (Thermo Fisher Scientific). Samples were resolved via NuPAGE 4–12% Bis-Tris Gels (Thermo Fisher Scientific) and immunoblotted overnight in transfer buffer.^50^ All blots were processed in parallel and derive from the same experiment. The dilutions of primary antibodies were as follows: AT8 (Thermo Fisher Scientific (MN1020)/PHF1 (a kind gift from Peter Davies, Albert Einstein College of Medicine) 1:10,000; AT180 (Thermo Fisher Scientific, MN1040) 1:5000; Tau5 (MAB-361)/Tau12 (MAB2241) obtained from Millipore and used at 1:20,000; SNAP-25 (a kind gift from the late Michael Wilson) 1:20,000; GAPDH (Millipore, AB2302) 1:20,000. Uncut blots can be found in Supplementary Fig. 4.

### Sarkosyl-insoluble preparation

The Sarkosyl-insoluble fraction of MAPT was isolated from hippocampal tissues as described previously^64^ with minor modifications, which have been previously described.^50^ Briefly, we processed the hippocampal tissue as indicated above, and then sonicated the insoluble pellet (P1) with 10 volumes of cold buffer H (10 mM Tris-HCl, 1 mM EGTA, 0.8 mM NaCl, 10% sucrose, pH 7.4) supplemented by 0.1 mM PMSF (Sigma-Aldrich), with phosphatase and protease inhibitor cocktails (Sigma Aldrich, P5726 and P8340, respectively). After centrifugation at 34,000×*g* in a Beckman Ti TLA-120.2 rotor for 30 min at 4 °C, the supernatant (S1) was adjusted to 1% (w/v) N-laurylsarcosine (Sigma-Aldrich) and 1% (v/v) 2-mercaptoethanol (Sigma-Aldrich) and incubated at 37 °C for 2 h with agitation. After centrifugation at 100,000 RPM for 35 min at room temperature, the Sarkosyl-soluble supernatant (S2) was collected and resuspended in 1× NuPAGE® LDS Sample Buffer (Thermo Fisher Scientific). Sarkosyl-insoluble pellet (P2) was washed several times in 1% sarkosyl solution, prepared in buffer H. The dilutions of primary antibodies (AT8 and Tau12) were similar to previously-stated dilutions.

### qPCR

RNA was extracted from the hemi-brain using the Trizol Reagent as described by the manufacturer and quantified via Nanodrop 2000. Total RNA was standardized (100 ng/μL) and converted to cDNA using the High Capacity cDNA Reverse Transcription kit and amplified using specific TaqMan probes (*Il1β*, Interleukin 1 beta, Mm00434228_m1; *TNFα*, Τumor necrosis factor alpha, Mm00443258_m1; *IL10*, Interleukin 10, Mm00439615_m1; *RETNLA*, Resistin-like alpha, Mm00445109_m1; *IL18*, Interleukin 18, Mm00434225_m1; *IFNγ*, Interferon gamma, Mm00801778_m1) and normalized to endogenous Mouse *GAPDH*, glyceraldehyde 3-phosphate dehydrogenase, on the StepOnePlus^®^ Life Technologies Real-Time PCR System (all reagents from Thermo Fisher Scientific).

### Mononuclear cell isolation and flow cytometry

Following a previously established technique,^51^ mononuclear cells were isolated from the brains of vaccinated animals, 1 week following the final vaccination, via density centrifugation between the 70%/30% Percoll (GE Healthcare technologies) interphase, washed with FACs buffer (5% FBS/PBS), and resuspended with FACs buffer. Cells were counted using an automatic cell counter (TC20 Automated Cell Counter, Bio-Rad) and cell counting slides (TC20 Dual-chamber slides, cat # 1450011) and then resuspended at a concentration of 500,000 cells/mL. Isolated cells were incubated with Fc block (CD16/CD32, BD Biosciences, Catalog #553141, used at 1:100), and subsequently incubated with antibodies (CD45 (conjugated to APC, BD Biosciences, catalog #559864, used at 1:100), CD11b (conjugated to FITC, Thermo Fisher Scientific, catalog #11–0112–41, used at 1:100), and CD3 (conjugated to PE, RnD, catalog #MAB4841, used at 1:100) for 1 h. Flow Cytometry was performed with a BD LSR Fortessa and quantified using FACs software (FACsDiva, BD Biosciences, USA). Gating strategies for populations analyzed and presented, can be found in Supplementary Fig. 5.

### Magnetic resonance imaging (MRI)

Animals were imaged using a 4.7 Tesla Biospec MRI scanner (Bruker Biospin; Billerica) equipped with a single-tuned surface coil for mouse brain. Briefly, mice were anesthetized with isoflurane gas (induction dosage 2–3%; maintenance dose 1.5–2%) with a mixture of O_2_:NO_2_ gases in the ratio of 2:1, delivered during the recording period, approximately 1–3 h). The following images were acquired: T2-weighted (T2_w_) images to assess gray matter integrity, specifically in the cortex (CX), hippocampal areas (HP, including entorhinal cortex), and corpus callosum (CC); T1-weighted (T1_w_) images to assess T1 relaxation time; and diffusion tensor imaging (DTI) was used to assess white matter integrity, including fractional anisotropy (FA), apparent diffusion coefficient (ADC, or mean diffusion, MD), radial diffusion (RD), and axial diffusion (AD) in all three brain regions. To assess volumetric changes, T2_w_ images were minimally processed, and using the spline tool on VivoQuant^TM^ (inviCRO, LLC, Boston, MA) and the Allen Brain Atlas as a reference (www.allenbrainatlas.org), regions of interest (ROI) were hand-drawn around the CX, HP, and CC on every MR brain slice (*n* = 18 slices, per mouse). Additional image processing and analysis were performed using Paravision (ver. 5.1, Bruker, Germany), the Bruker plugin available on FIJI Image J (NIH, USA), and VivoQuant software.

### Novel object recognition task (NOR)

On the first day of this 3-day paradigm, animals were acclimated to an open 75 cm^2^ arena for 5 min. Twenty-four hours later, the mice were exposed to two identical objects (2 glass jars) for 5 min. Twenty-four hours later, the mice were exposed to one of the previous familiar objects (glass jar) and a novel object (plastic water bottle). The percentage of time spent with the novel object vs. the familiar object was recorded as a measurement of delay-dependent memory.^65^ Results presented were combined data from two independent experiments, acquired with Ethovision XT8 (Noldus, Netherlands), and then processed in Excel and Prism.

### Morris water maze task (MWM)

Animals were randomized to a blinded cage labeled with a number using a freely available random number generator (www.random.org) so that the experimenter was blinded to the genotype and treatment of the animal. Then these animals were trained for 5 days (4 daily trials, approximately 25 min inter-trial interval) to find a hidden platform submerged beneath milky water (26 °C) using spatial cues on the wall, as previously described.^66,67^ Briefly, during the acquisition phase, mice navigate in a tank of opaque water for a period of 1 min, and over 5 days (four trials daily) learn to navigate to a submerged platform using visual cues on the wall (each visual cue was approximately 2′ × 4′ and were made with different black/white shapes so that each visual cue was distinctly different). Animals were randomized to search for a platform in a different area of the MWM so results were counter-balanced; no bias was present to one quadrant over others. Animals were singly housed and kept warm in cages between trials. On the sixth day, a probe trial was performed via removal of the platform and the percentage of time spent in the target quadrant vs. the other quadrants was recorded as a measurement of working spatial memory. Results presented were combined data from two independent experiments, and all behavioral data were collected using Ethovision XT8 software (Noldus, Netherlands), and then processed in Excel and Prism.

### Statistical analysis and study blinding

Animal numbers for all experiments were determined by doing a Power analysis. Statistical analysis was performed using GraphPad Prism (USA) software. A Student’s *t*-test was employed when two groups were being analyzed. A one-way ANOVA, with a Tukey’s post-hoc test, was used when investigating three or more groups. Two-way multiple comparisons ANOVA was used for analysis of the Morris water maze learning curve.

All experiments were performed by using unique pathology numbering system (for necropsy studies) to avoid any subjective bias. Experimenter blinded to the genotype and treatment performed for all the experiments in this study. Genotype and treatment information was made available only after the completion of the analysis.

## Supplementary information

Supplementary Figures

Supplementary Movie 1

Supplementary Movie 2

## Data Availability

The datasets used and/or analyzed in the current study are available from the corresponding author upon reasonable request.

## Supplementary Information

**Supplementary Information** accompanies the paper on the *npj Vaccines* website (10.1038/s41541-019-0118-4).
