# Supplementary material for: Qß Virus-like particle-based vaccine induces robust immunity and protects against tauopathy
Source: NPJ Vaccines. 2019 Jun 3;4:26. doi: 10.1038/s41541-019-0118-4 (PMC6547647; doi:10.1038/s41541-019-0118-4)
Supplement: Supplementary file 1 — Supplementary Figures [file 41541_2019_118_MOESM1_ESM.docx]

**Qß Virus-like particle-based vaccine induces robust immunity and protects against tauopathy**

**Running title:** pT181-Qß vaccine protects against tauopathy

**Authors:**

Nicole M. Maphis^1^, Julianne Peabody^1^, Erin Crossey^1,†^, Shanya Jiang^1^, Fadi A. Jamaleddin Ahmad^6^, Maria Alvarez^6^, Soiba Khalid Mansoor^6^, Amanda Yaney^6^, Yirong Yang^3^, Laurel O. Sillerud^4^, Colin M. Wilson^5^, Reed Selwyn^5^, Jonathan L. Brigman^2^, Judy L. Cannon^1^, David S. Peabody^1^, Bryce Chackerian^1^ and Kiran Bhaskar^1,4^*

**Affiliations:**

^1^Department of Molecular Genetics and Microbiology, University of New Mexico, Albuquerque NM 87131 USA.

^2^Department of Neurosciences, University of New Mexico, Albuquerque NM 87131 USA.

^3^College of Pharmaceutical Sciences, University of New Mexico, Albuquerque NM 87131 USA.

^4^Department of Neurology, University of New Mexico, Albuquerque NM 87131 USA.

^5^Department of Radiology, University of New Mexico, Albuquerque NM 87131 USA.

^6^School of Medicine, University of New Mexico, Albuquerque NM 87131 USA.

**Supplemental Information:** 5 figures and 1 table

**Supplementary Figure 1.** Gallyas silver impregnation reveals significant decreases in the number of neurofibrillary tangles (NFTs) following pT181-Qß immunization in rTg4510.

**Supplementary Figure 2.** qRT-PCR gene expression is unaltered by pT181-Qß vaccination in the rTg4510 mouse model of tauopathy.

**Supplementary Figure 3.** Additional Novel Object and Morris Water Maze Behavioral Data.

**Supplementary Figure 4.** Uncut Blots/Gels.

**Supplementary Figure 5.** Flow cytometry gating strategy for data in **Fig 5**.

**Supplementary Table 1.** Descriptive Statistics for T2 and Diffusion Weighted Imaging (DWI) of the entire brain reveals no age-related diffusivity changes in pT181-Qßvs. Qß-treated rTg4510.

**
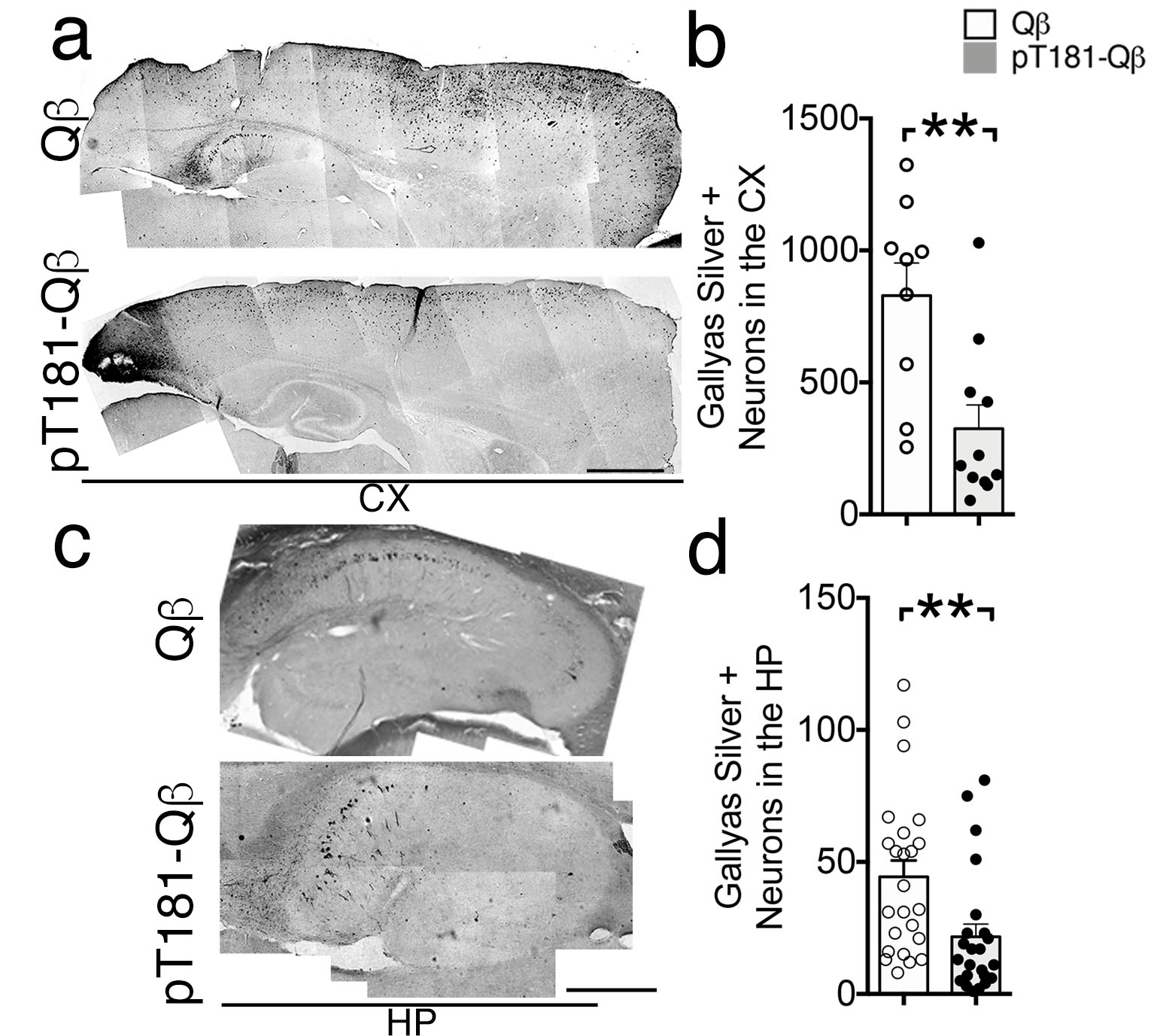
**

**Supplementary Figure 1. Gallyas silver impregnation reveals significant decreases in the number of neurofibrillary tangles (NFTs) following pT181-Qß immunization in rTg4510.**

Quantitative morphometry was performed on at least 3 stained sections per animal, at similar sagittal planes, in each group using the hand-drawn ROI tool in FIJI Image J (NIH, USA) and the thresholding tool. The analysis revealed significant decreases in the number of neurons positive for NFTs in the CX (**a,** where approximately 12 images taken at 10x magnification were stitched together, quantified in **b**) and the HP (**c,** where approximately 6 images taken at 20x magnification were stitched together, quantified in **d**). All graphs display mean ± SEM, significance values were determined with a student’s *t*-test (p ≤ 0.01 **). Scale bar in **a** is 2 mm and in **c** is 1 mm.

**
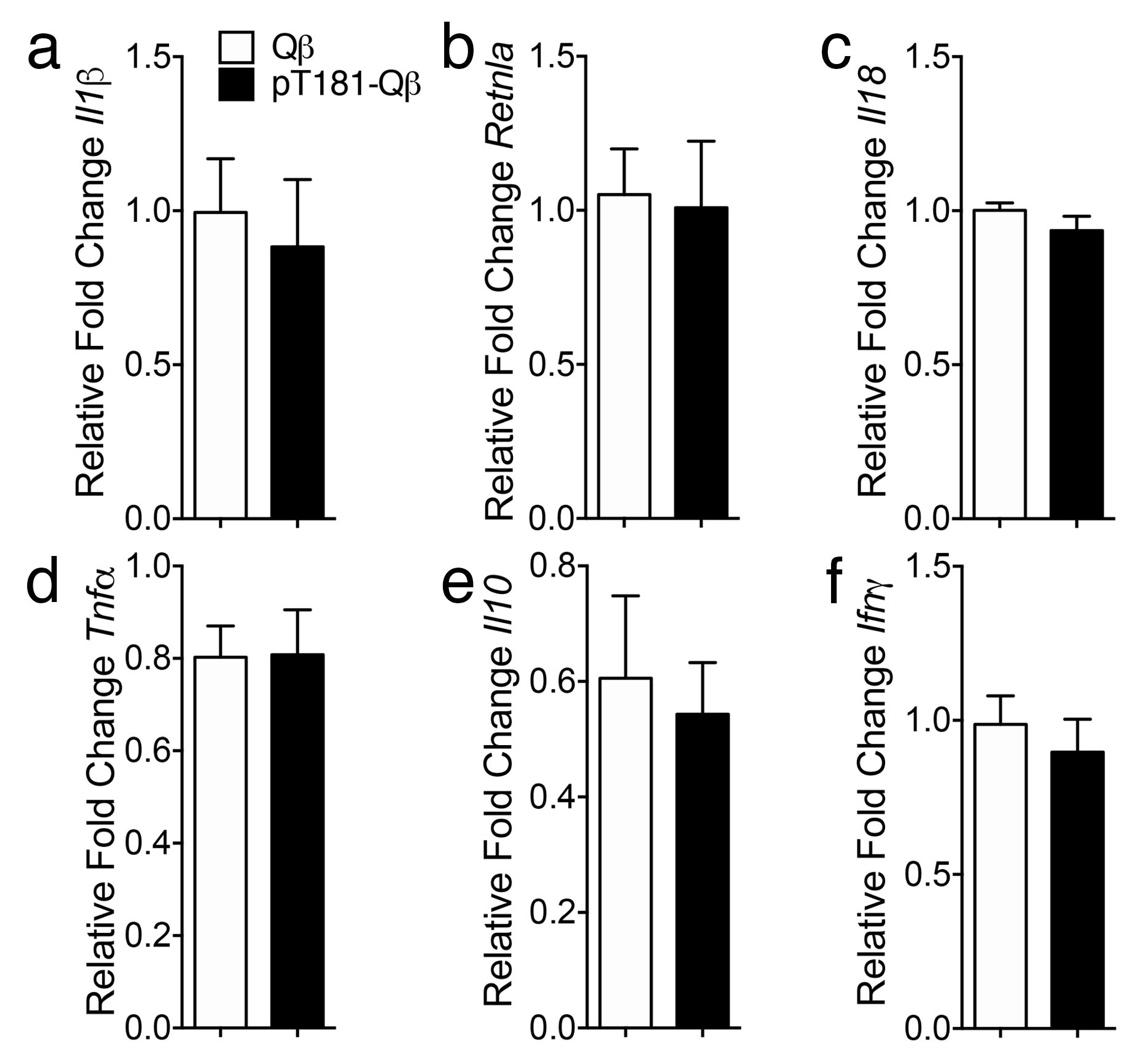
**

**Supplementary Figure 2. pT181-Qß vaccination in the rTg4510 mouse model of tauopathy does not alter the inflammatory profile of the brain.** mRNA was isolated by TRIZOL from snap frozen brain tissue, reverse transcribed into cDNA and then measured using readily available assays from Thermo Fisher Scientific by real time PCR. There were no observed differences in mRNA of common pro-inflammatory genes (*Il1β* **a**, and *Tnfα* **d**), anti-inflammatory genes (*Retnla* **b**, and *Il10* **e**), or T-cell related genes (*IL18* **c**, and *Ifnγ* **f**). All graphs display mean ± SEM, significance values were determined with a student’s *t*-test


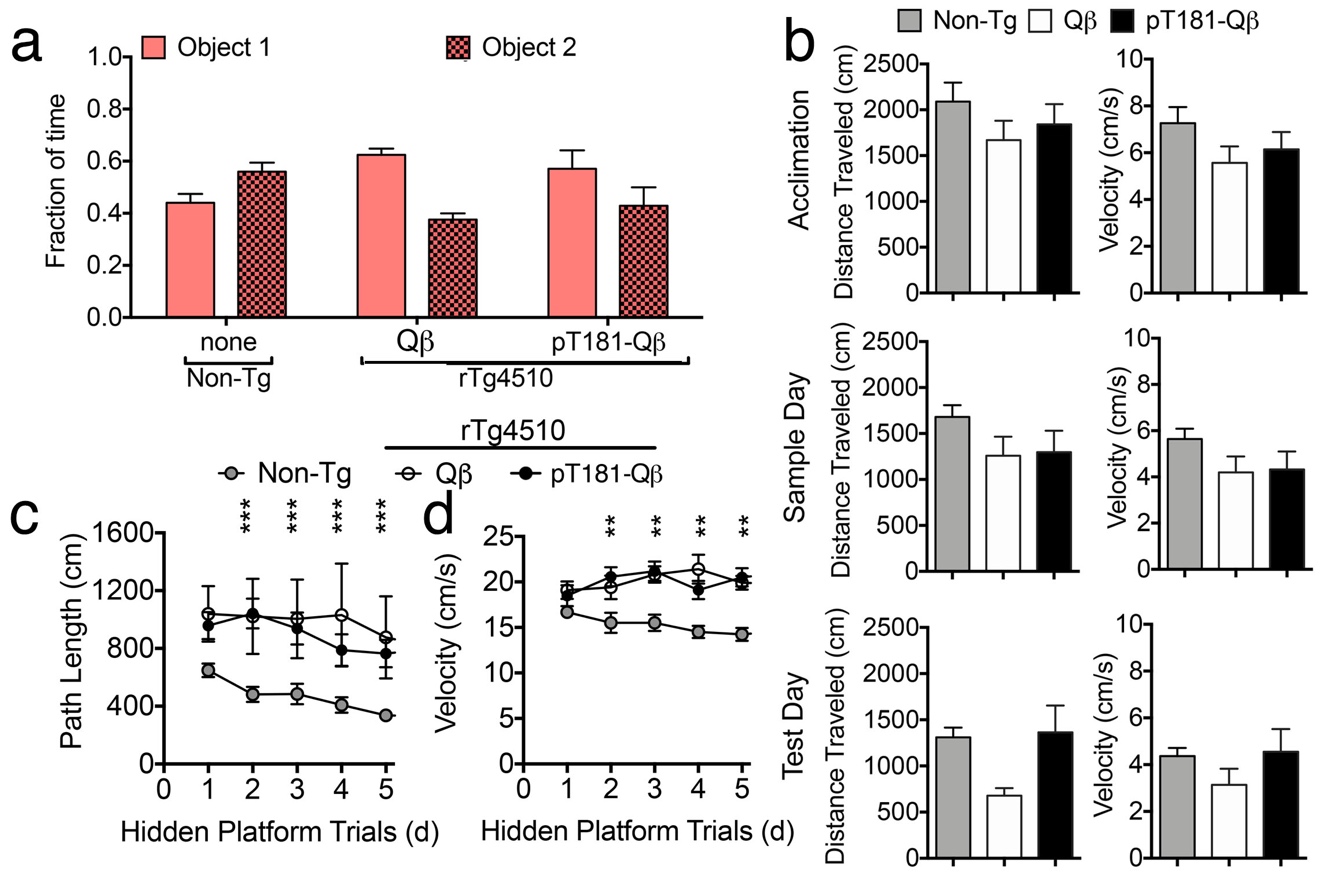


**Supplementary Figure 3.** **Additional Novel Object and Morris Water Maze Behavioral Data.** Neither group displayed a preference for either identical object (object 1 or 2) during day two (Sample Day) of the Novel Object Recognition Task (NOR) (A). There were no significant differences in distance traveled or velocity between Non-Tg or rTg4510, regardless of vaccination, during acclimation, sample or test days (B). Non-Tg had a significantly shorter path length (C) and swam with a slower velocity (D) during the hidden platform trials, which was not affected by vaccination in the rTg4510. All graphs display mean ± SEM, significance values were determined with a Two-Way ANOVA (**a**), One-Way ANOVA (**b**) or with a Repeated Measures Two-Way ANOVA (**c-d**), p ** < 0.01, ***< 0.001).

**Supplementary Figure 4. Uncut gels/blots**

**Figure 1b.** * shows the lanes displayed.

**
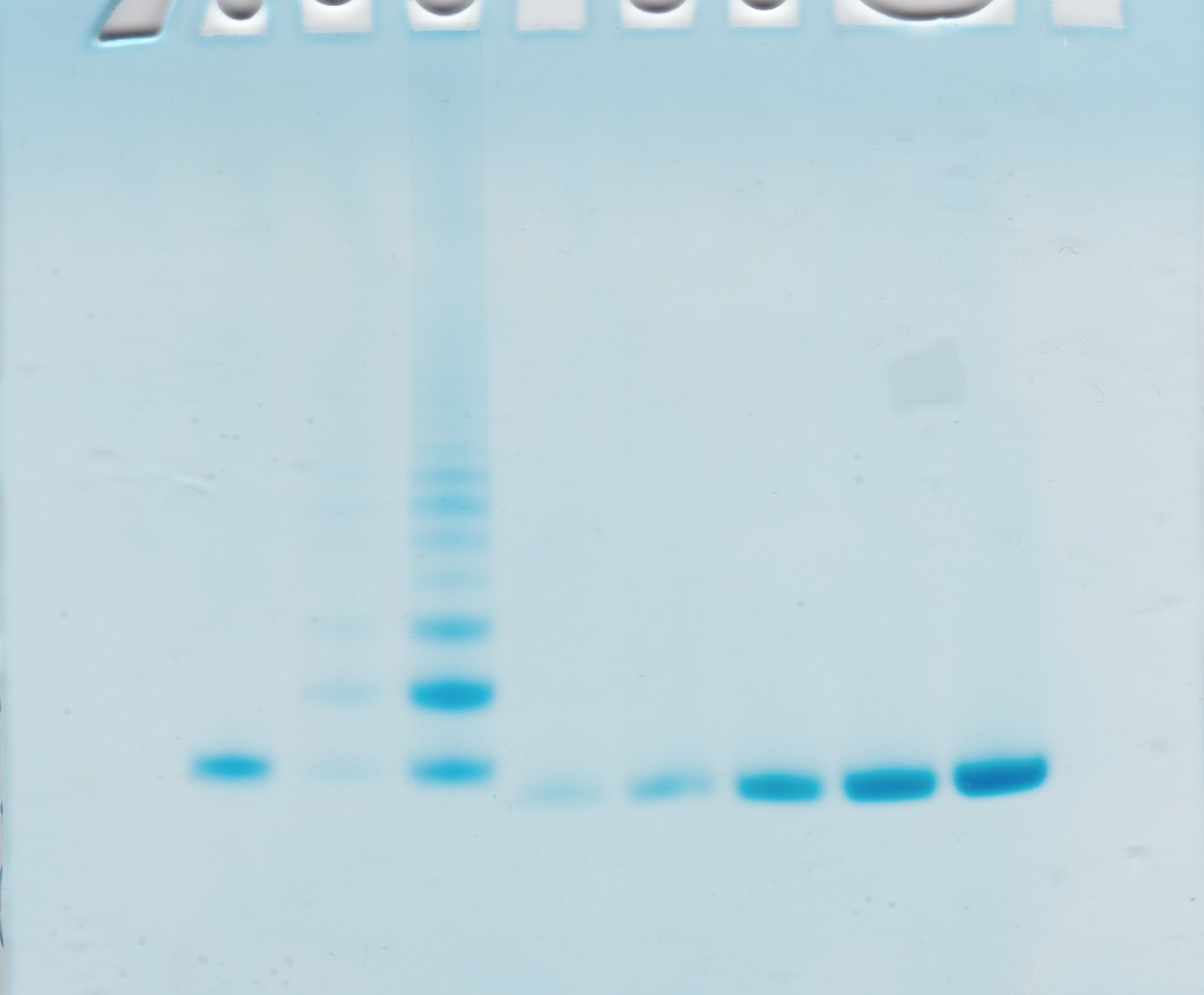
**

*

*

**Figure 4a.**

**AT8 AT180 Tau5 GAPDH**

**
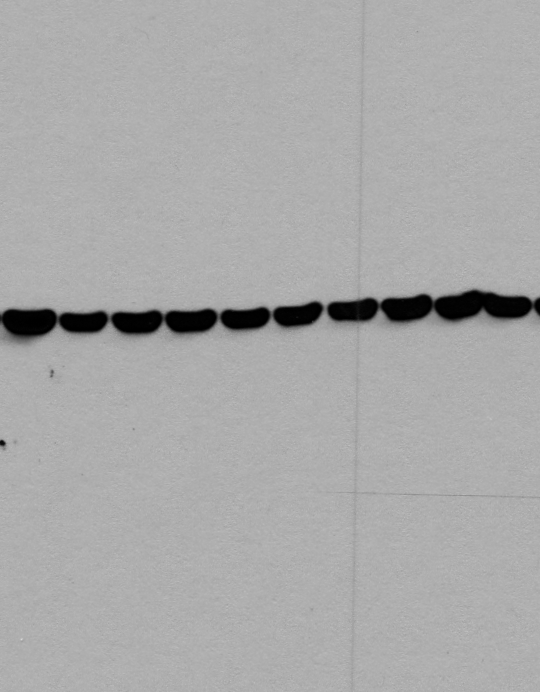
**

**
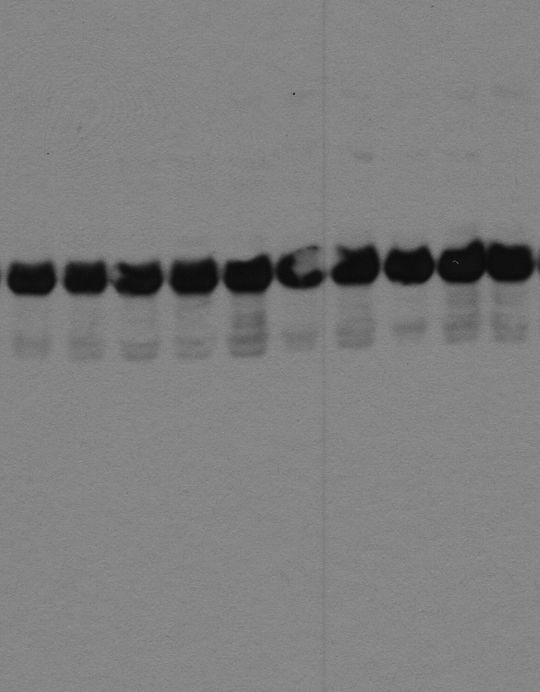

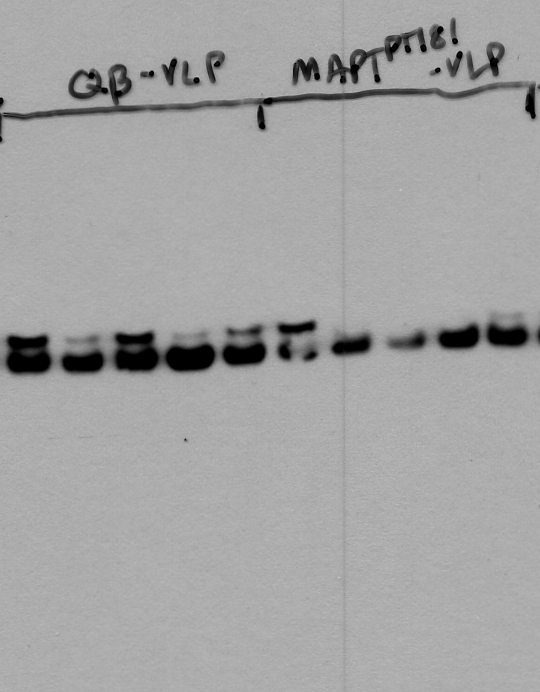

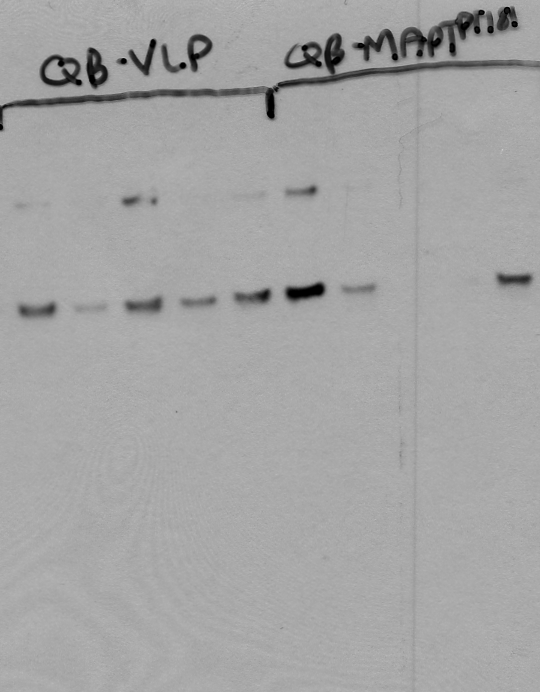
**

**Figure 4g.**

**Sarkosyl insoluble fraction: Sarkosyl soluble fraction**

**
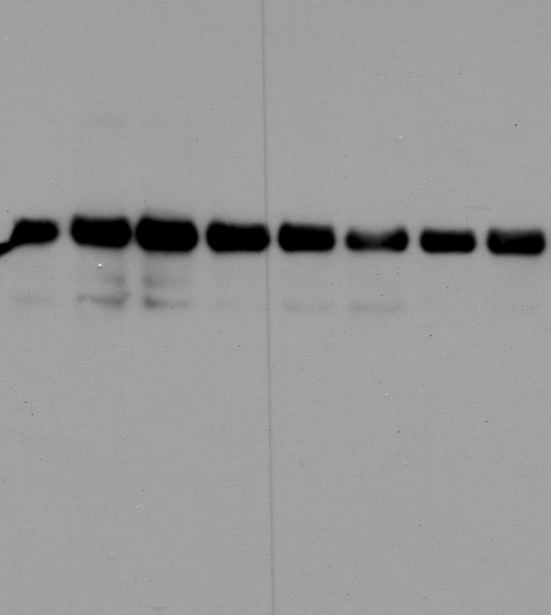
AT8 Tau 12 AT8 Tau12**

**
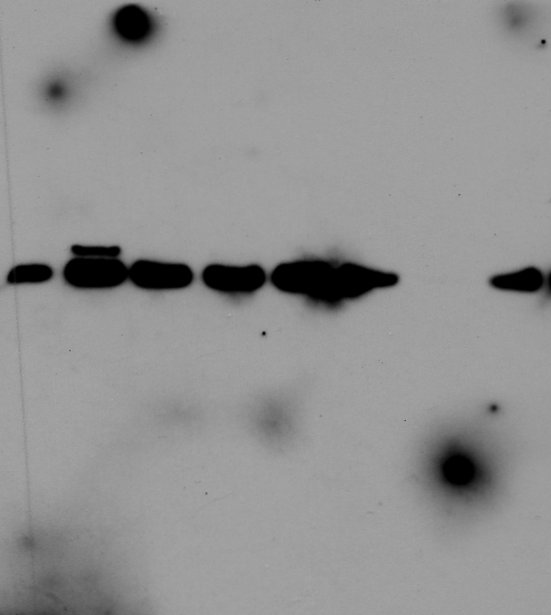

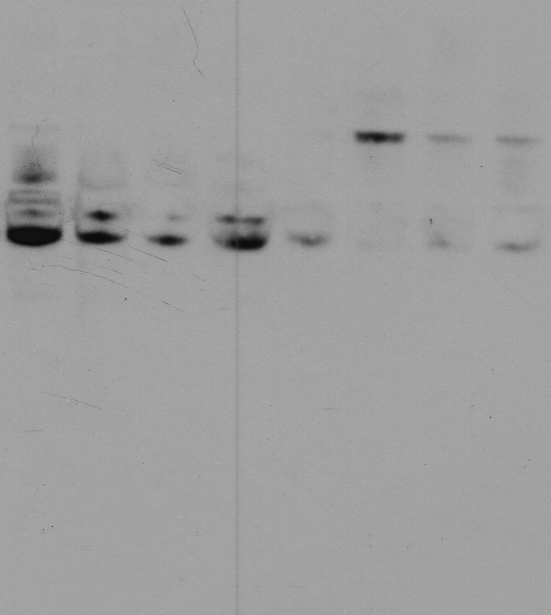

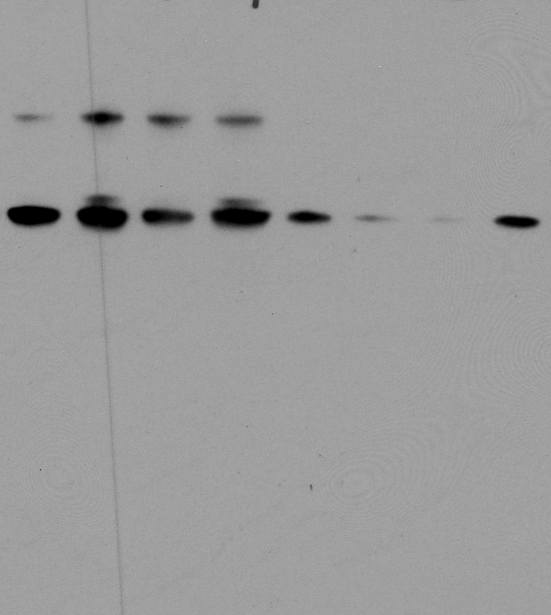
**

**Figure 6k/l Qß pT181-Qß**

**
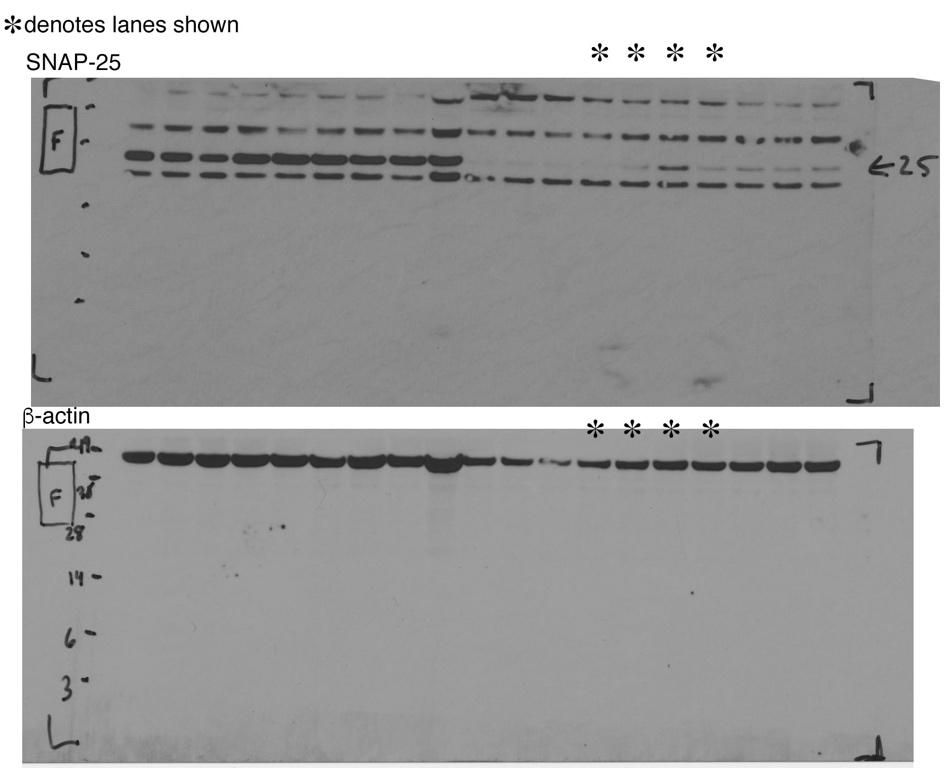
**


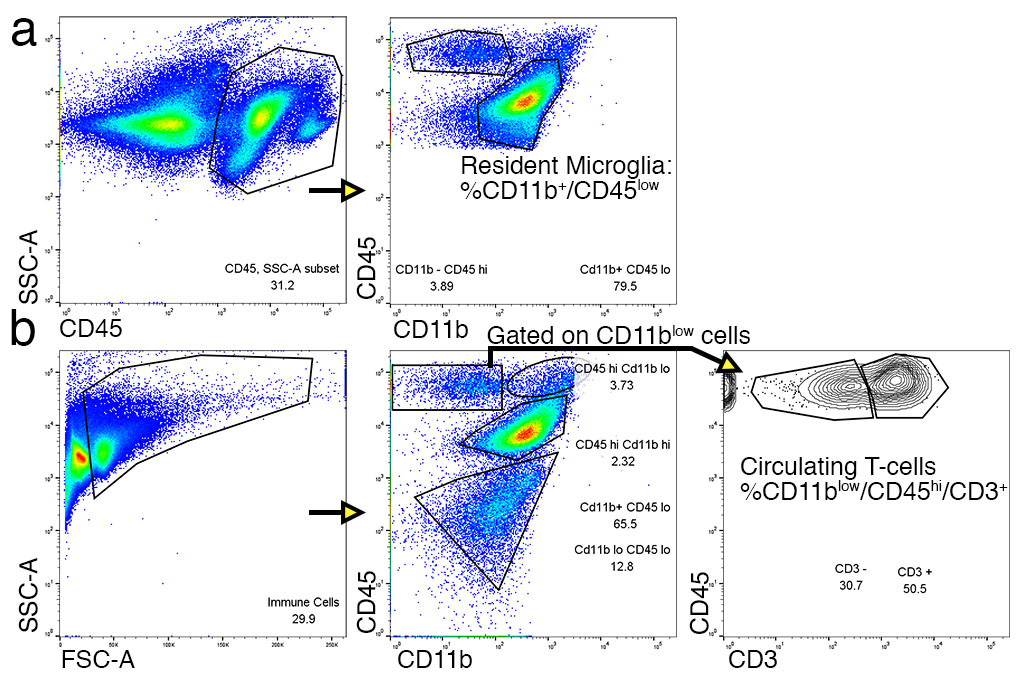


**Supplementary Figure 5.** Gating strategies used for cell sorting. (a) Gating strategy used to sort the percentage of resident microglia (CD11b+/CD45 low cells) present in isolated mononuclear cells (MNCs) of rTg4510 mice receiving either Qß or pT181-Qß vaccination and presented in **Fig. 5e** in green**.** (b) Gating strategy used to sort out the percentage of circulating T-cells present (% CD11b low/CD45 hi/CD3+ cells) in isolated MNCs of rTg4510 mice receiving either Qß or pT181-Qß vaccination and presented in **Fig. 5f** in red. Data shown is from MNCs isolated from a Qß-vaccinated rTg4510 mouse.

|  |  | pT181-Qβ (n=8) | Qβ (n=9) |  |
| --- | --- | --- | --- | --- |
|  |  | mean +/- SD | mean +/- SD | p value |
| T2 (x10^6^) | CX | 3.24 +/- 0.32 | 3.12 +/- 0.29 | 0.428 |
|  | HP | 2.57 +/- 0.22 | 2.50 +/- 0.19 | 0.500 |
|  | CC | 2.56 +/- 0.26 | 2.42 +/- 0.19 | 0.264 |
| MD (x10^4^) mm^2^/sec | CX | 7.99 +/- 0.41 | 8.12 +/- 0.62 | 0.619 |
|  | HP | 8.40 +/- 0.36 | 8.45 +/- 0.38 | 0.786 |
|  | CC | 8.20 +/- 0.46 | 8.26 +/- 0.44 | 0.785 |
| AD (x10^4^) mm^2^/sec | CX | 9.89 +/- 0.71 | 10.1 +/- 0.90 | 0.547 |
|  | HP | 11.3 +/- 0.62 | 11.3 +/- 0.56 | 0.972 |
|  | CC | 11.6 +/- 0.68 | 11.4 +/- 0.69 | 0.590 |
| RD (x10^4^) mm^2^/sec | CX | 7.10 +/- 0.38 | 7.09 +/- 0.48 | 0.961 |
|  | HP | 6.96 +/- 0.27 | 7.02 +/- 0.30 | 0.689 |
|  | CC | 6.51 +/- 0.38 | 6.71 +/- 0.38 | 0.291 |
| T1 (ms) | CX | 2541.11 +/- 84.12 | 2514.52 +/- 148.71 | 0.664 |
|  | HP | 2594.87 +/- 111.45 | 2503.66 +/- 156.72 | 0.196 |
|  | CC | 2480.11 +/- 102.07 | 2377.93 +/- 143.91 | 0.120 |
| FA | CX | 0.23 +/- 0.03 | 0.24 +/- 0.02 | 0.686 |
|  | HP | 0.31 +/- 0.02 | 0.31 +/- 0.01 | 0.711 |
|  | CC | 0.39 +/- 0.03 | 0.37 +/- 0.03 | 0.690 |

Supplementary Table 1. Descriptive Statistics for T2 and Diffusion Weighted Imaging (DWI) of the entire brain reveals no treatment-related diffusivity changes in pT181-Qß vs. Qß-treated rTg4510. There were no significant differences in mean T2 signal, Mean Diffusivity (MD, mm^2^/s), Axial Diffusivity (AD, mm^2^/s), Radial Diffusivity (RD, mm^2^/s), T1 relaxation time (milliseconds), or Fractional Anisotropy (FA, no units) in the cortex (CX), hippocampus (HP), or Corpus Callosum (CC) in pT181-Qß vaccination compared with Qß alone in the rTg4510 mice. Table displays mean ± SD, significance values were determined with a student’s *t* test and were not significant n = 8 (Qß) n = 9 (pT181-Qß).
